# Negative Regulation of Type I IFN Expression by OASL1 Permits Chronic Viral Infection and CD8^+^ T-Cell Exhaustion

**DOI:** 10.1371/journal.ppat.1003478

**Published:** 2013-07-18

**Authors:** Myeong Sup Lee, Chan Hee Park, Yun Hee Jeong, Young-Joon Kim, Sang-Jun Ha

**Affiliations:** 1 Genome Research Center, Department of Biochemistry, College of Life Science and Biotechnology, Yonsei University, Seoul, Republic of Korea; 2 System Immunology Laboratory, Department of Biochemistry, College of Life Science and Biotechnology, Yonsei University, Seoul, Republic of Korea; 3 Department of Integrated Omics for Biomedical Science, WCU Program of Graduate School, Yonsei University, Seoul, Republic of Korea; University of Pennsylvania, United States of America

## Abstract

The type I interferons (IFN-Is) are critical not only in early viral control but also in prolonged T-cell immune responses. However, chronic viral infections such as those of human immunodeficiency virus (HIV) and hepatitis C virus (HCV) in humans and lymphocytic choriomeningitis virus (LCMV) in mice overcome this early IFN-I barrier and induce viral persistence and exhaustion of T-cell function. Although various T-cell-intrinsic and -extrinsic factors are known to contribute to induction of chronic conditions, the roles of IFN-I negative regulators in chronic viral infections have been largely unexplored. Herein, we explored whether 2′–5′ oligoadenylate synthetase-like 1 (OASL1), a recently defined IFN-I negative regulator, plays a key role in the virus-specific T-cell response and viral defense against chronic LCMV. To this end, we infected *Oasl1* knockout and wild-type mice with LCMV CL-13 (a chronic virus) and monitored T-cell responses, serum cytokine levels, and viral titers. LCMV CL-13-infected *Oasl1* KO mice displayed a sustained level of serum IFN-I, which was primarily produced by splenic plasmacytoid dendritic cells, during the very early phase of infection (2–3 days post-infection). *Oasl1* deficiency also led to the accelerated elimination of viremia and induction of a functional antiviral CD8 T-cell response, which critically depended on IFN-I receptor signaling. Together, these results demonstrate that OASL1-mediated negative regulation of IFN-I production at an early phase of infection permits viral persistence and suppresses T-cell function, suggesting that IFN-I negative regulators, including OASL1, could be exciting new targets for preventing chronic viral infection.

## Author Summary

Chronic viral infections, such as those of human immunodeficiency virus (HIV) and hepatitis C virus (HCV) in humans, remain serious health problems worldwide, necessitating alternative targets/reagents for better treatment. Although the production of and/or response to type I interferon (IFN-I), a critical antiviral reagent, are known to be dysregulated in chronic viral infections, no serious effort has been performed to determine whether any host IFN-I negative regulator can importantly contribute to inducing and/or maintaining chronic viral infections. In this study, we used a mouse model of chronic viral infection, lymphocytic choriomeningitis virus (LCMV) infection, and asked whether 2′–5′ oligoadenylate synthetase-like 1 (OASL1), a recently defined IFN-I negative regulator, plays a key role in the viral defense against chronic LCMV infection. Our data show that OASL1 suppresses IFN-I production during very early phase of infection, thus inhibits efficient viral control and the induction of functional virus-specific T-cell response, permitting viral persistence. These results indicate that OASL1-mediated suppression of IFN-I production is a critical step for permitting chronic viral infection and suggest that IFN-I negative regulators, including OASL1, could be exciting new targets for preventing chronic viral infection.

## Introduction

Pattern-recognition receptors (PRRs) displayed on innate immune cells such as dendritic cells (DCs) and macrophages (Macs) sense pathogens by recognizing conserved pathogen-associated molecular patterns (PAMPs) [1], [2]. Major trans-membrane PRRs that sense viruses are the Toll-like receptors (TLRs) such as TLR3, TLR7, and TLR9, and such cytosolic PRRs are retinoic acid-inducible gene I (RIG-I)-like receptors (RLRs) such as RIG-I and melanoma differentiation-associated gene 5 (MDA5) [3]. Upon recognition of cognate ligands, these PRRs initiate various signaling pathways that lead to the production of inflammatory cytokines, including type I interferons (IFN-Is), such as IFN-αs/β, which are critical for inhibiting early viral replication in the host [3], [4]. Additionally, antigen-presenting cells (APCs), particularly DCs, up-regulate co-stimulatory molecules and major histocompatibility complex (MHC) molecules upon viral sensing and induce the differentiation of effector T cells which are key adaptive immune cells required for later viral clearance [5], [6].

The host immune system can effectively induce virus-specific T-cell activation, expansion, and successful generation of memory T cells upon acute viral infections. However, the immune system cannot induce such response upon chronic viral infections such as those of human immunodeficiency virus (HIV), hepatitis C virus (HCV), or Epstein Barr virus (EBV) in humans, and lymphocytic choriomeningitis virus (LCMV) in mice. As a result, the hosts live with a persistent viral load throughout their life-spans and have fundamentally dysfunctional T cells that produce dampened effector cytokines [7], [8]. Various virus-specific T-cell-intrinsic and -extrinsic factors have been known to contribute to inducing and/or maintaining the chronic conditions. Virus-specific T-cell-intrinsic factors include elevated expression of inhibitory receptors such as programmed death-1 (PD-1), T-cell immunoglobulin and mucin protein-3 (TIM-3), cytotoxic T-lymphocyte antigen-4 (CTLA-4), and lymphocyte-activation gene-3 (LAG-3) [9]–[14], whereas virus-specific T-cell-extrinsic factors include altered antigen presentation by impaired DCs [15], enhanced immune suppression by regulatory T (Treg) cells [16], [17], and increased immunosuppressive cytokines such as interleukin-10 (IL-10) [18], [19] and transforming growth factor-β (TGF-β) [20], [21].

In addition, suppression of IFN-I production and response could be a major contributing factor leading to the chronic condition. Suppression may be a result of the reduced number of plasmacytoid DCs (pDCs), a major cellular source of IFN-I upon various viral infections. Indeed, the numbers of pDCs are reduced in humans and mice during infections with HIV, HCV, EBV, and LCMV [22]–[27]. Alternatively, such suppression could be actively regulated by host- and virus-derived negative factors that act on diverse PRR-signaling components and two major transcription factors (TFs), interferon regulatory factor 3 (IRF3) and IRF7 [28]–[30], involved in IFN-I production as well as on IFN-I receptor signaling components involved in the IFN-I response [28]–[30]. To overcome the dysregulated IFN-I production, IFN-I has been used clinically to treat patients infected with certain chronic viruses such as HCV. However, high dose and long-lasting IFN-I treatment is known to be necessary to achieve any therapeutic benefit, implying that there are some underlying mechanisms to negatively regulate the IFN-I response [31]. Therefore, whether host-derived IFN-I negative regulator plays any significant role in promoting viral persistence in the setting of chronic viral infection is quite an important question.

Recently, we showed that 2′–5′ oligoadenylate synthetase (OAS)-like 1 (OASL1), an IFN-stimulated gene (ISG), is a novel translation inhibitor of IRF7, the IFN-inducible IFN-I master TF, and negatively regulates robust IFN-I production during acute viral infections [32]. Therefore, we hypothesized that OASL1 could suppress IFN-I production during chronic LCMV infection by inhibiting IRF7 production and permit persistent viral infection. In the present study, we show that *Oasl1* knockout (KO) mice displayed a sustained level of IFN-I during early viral infection with LCMV clone 13 (LCMV CL-13, a chronic virus), controlled viremia quickly and induced better functional T-cell responses compared with wild-type (WT) mice. In addition, we show that IRF7 protein expression was higher in virus-infected *Oasl1* KO spleen than in WT spleen and that IFN-I receptor signaling during this early period was necessary for accelerated viral control and the enhanced T-cell immune response in the *Oasl1* KO mice. These results indicate that OASL1-mediated negative regulation of IFN-I production at the early phase of the infection is critical in permitting viral persistence.

## Results

### An accelerated control of viremia and a better virus-specific CD8^+^ T-cell differentiation in *Oasl1* KO mice upon chronic LCMV infection

**Figure 1:**
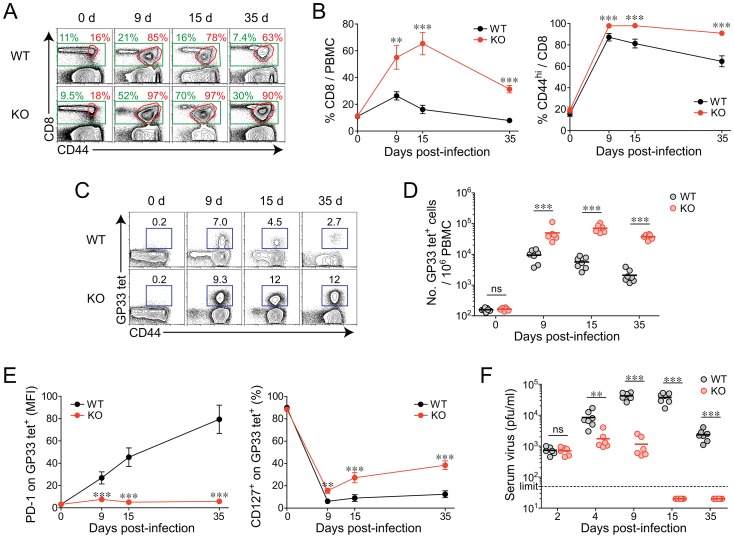
Rapid expansion and maintenance of virus-specific CD8^+^ T cells and accelerated viral control in *Oasl1* KO mice during chronic LCMV infection. WT and *Oasl1* KO mice were infected with LCMV CL-13. (**A–E**) PBMCs were collected at indicated time points p.i. and analyzed by flow cytometry. (**A**) Representative data showing CD8^+^ T-cell percentage (green) among lymphocytes and CD44^hi^ cell percentage (red) among CD8^+^ T cells. (**B**) Summary showing CD8^+^ T-cell percentage among lymphocytes and CD44^hi^ cell percentage among CD8^+^ T cells. (**C**) Representative data showing the frequency of GP_33–41_ (GP33) tetramer-positive cells among CD8^+^ T cells. Numbers in plots indicate percentage of the tetramer-positive cells. (**D**) The numbers of GP33 tetramer-positive CD8^+^ T cells per 10^6^ PBMCs during the course of LCMV CL-13 infection. (**E**) Summary showing the PD-1 expression level on GP33 tetramer-positive CD8^+^ T cells as represented by mean fluorescence intensity (MFI), and the percentage of CD127^+^ cells among GP33 tetramer-positive CD8^+^ T cells. (**F**) Serum viral titer in WT and *Oasl1* KO mice at the indicated time p.i. Dashed black line represents the virus detection limit. All line graphs show mean ± standard deviation (SD). Data are representative of four independent experiments (*n*>6 per group in each experiment). ns, not significant; **, *P*<0.01; ***, *P*<0.001.

To investigate the *in vivo* role of OASL1 in T-cell differentiation and viral defense upon chronic viral infection, we infected both WT and *Oasl1* KO mice with LCMV CL-13 and monitored CD8^+^ T-cell numbers and phenotypes as well as viral titers in the blood for 35 days (d) post-infection (p.i.). The percentage of total CD8^+^ T cells in peripheral blood mononuclear cells (PBMCs) became much higher in KO mice than in WT mice (>3-fold at 15 d p.i.) upon infection (Fig. 1A and 1B). In addition, the frequency of activated CD8^+^ T cells expressing CD44 was maintained at a slightly higher percentage in KO mice than in WT mice after infection (Fig. 1A and 1B). The CD44-expressing cells in *Oasl1* KO mice at 35 d p.i. were mostly specific to various LCMV epitopes (approximately 80% of CD44^+^ cells) (data not shown). Notably, the number of LCMV GP_33–41_ (GP33) peptide-specific CD8^+^ T cells was more strongly increased in KO mice compared with WT mice (>10-fold at 15 d p.i., Fig. 1C and 1D). These results indicate that virus-specific CD8^+^ T cells undergo much more massive expansion in *Oasl1* KO mice compared with WT mice.

Expression of PD-1 on GP33 peptide-specific CD8^+^ T cells did not appear to be significantly induced in *Oasl1* KO mice after LCMV CL-13 infection, although WT mice exhibited strongly induced PD-1 expression patterns (Figs. 1E and S1A). CD127, a marker of memory T cells, was progressively up-regulated in KO mice but not in WT mice after initial downregulation (Figs. 1E and S1B). These data imply that CD8^+^ T-cell responses might also be better qualitatively in *Oasl1* KO mice upon chronic viral infection. Remarkably, there was complete control of viremia in serum at 15 d p.i. in LCMV CL-13-infected *Oasl1* KO mice, but viremia remained continuously high in WT mice serum by 35 d p.i. (Fig. 1F). Consistent with accelerated viral control in KO mice, the mice recovered rapidly from an initial loss in body weight, whereas WT mice did not recover after an even longer period of time (Fig. S2). As expected, *Oasl1* KO mice also controlled LCMV Armstrong (LCMV Arm, an acute strain of LCMV) infection much more quickly than WT mice (Fig. S3). Interestingly, the viral titers in *Oasl1* KO mice at 4 and 9 d p.i. upon LCMV CL-13 infection were quite comparable to those in LCMV Arm-infected WT mice (Fig. S3). The observation that *Oasl1* KO mice control the LCMV CL-13 infection as quickly as the WT mice control the LCMV Arm infection and the known fact that T-cell differentiation occurs efficiently in WT mice upon LCMV Arm infection [33], [34], together imply that rapid viral control in the blood of *Oasl1* KO mice upon LCMV CL-13 infection may be one of the major contributing factor for the enhanced CD8^+^ T-cell differentiation in the KO mice.

### Efficient induction of functional virus-specific T cells in tissues and complete control of LCMV CL-13 in *Oasl1* KO mice during the late phase of infection

**Figure 2:**
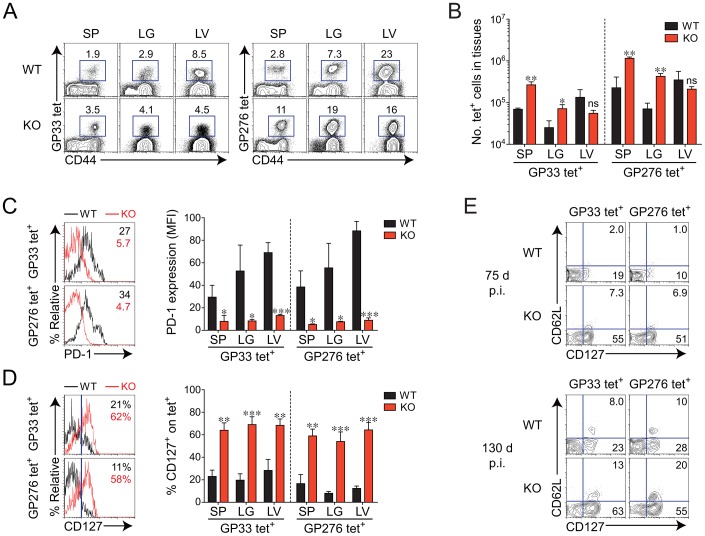
Phenotypic change of virus-specific CD8^+^ T cells in tissues of *Oasl1* KO mice during late phase of chronic LCMV infection. (**A**–**D**) Lymphocytes were isolated from the spleen (SP), lung (LG), and liver (LV) of LCMV CL-13-infected WT and *Oasl1* KO mice at 75 d p.i. and analyzed by flow cytometry. (**A**) Representative data showing the frequency of GP33 and GP_276–286_ (GP276) tetramer-positive cells among CD8^+^ T cells. (**B**) Absolute numbers of the tetramer-positive CD8^+^ T cells in the indicated tissues. (**C**, **D**) PD-1 and CD127 expression levels on virus-specific CD8^+^ T cells in the spleen of WT and KO mice. Numbers in the plots indicate PD-1 MFI value (**C**) on GP33 and GP276 tetramer-positive CD8^+^ T cells and CD127^+^ cell percentage (**D**) among the tetramer-positive cells. Blue vertical lines in the plots of (**D**) divide CD127^+^ and CD127^−^ tetramer-positive CD8^+^ T cells. The PD-1 MFI values and CD127^+^ cell frequencies in the indicated tissues are also summarized in the graphs. (**E**) Co-expression pattern of CD127 and CD62L on virus-specific CD8^+^ T cells *ex vivo*. Splenocytes isolated from LCMV CL-13-infected WT and KO mice at 75 d p.i. (top) and 130 d p.i. (bottom) were analyzed by flow cytometry. GP33 and GP276 tetramer-positive CD8^+^ T cells were plotted: the numbers in the plots indicate percentages of CD127^+^CD62L^+^ (central memory) and CD127^+^CD62L^−^ (effector memory). All bar graphs show mean + SD. Data are representative of at least two independent experiments (*n* = 3–4 per group in each experiment). ns, not significant; *, *P*<0.05; **, *P*<0.01; ***, *P*<0.001.

We next asked whether the enhanced immune features observed in the blood from *Oasl1* KO mice were also present in tissues at a later phase of infection (more than 2 months p.i.). We included another tetramer specific to GP_276–286_ (GP276), an immunodominant epitope other than GP33, to exclude the possibility that the T-cell responses were virus epitope-specific phenomena. Frequencies and numbers of both GP33- and GP276-specific CD8^+^ T cells were significantly higher at 75 d p.i. in the spleen and lung of *Oasl1* KO mice than in those of WT mice, whereas the frequencies and numbers of such T cells seemed to be lower in the liver of KO mice (Fig. 2A and 2B). The observation that a slightly lower number of virus-specific T cells in the liver (a nonlymphoid tissue) of *Oasl1* KO mice is not unusual because frequent antigen exposure to T-cell receptor of CD8^+^ T cells in the condition of chronic infection is known to enable virus-specific CD8^+^ T cells to migrate preferentially into nonlymphoid tissues rather than lymphoid tissues [33]. However, PD-1 expression levels in GP33- and GP276-specific CD8^+^ T cells were significantly lower in the spleen, lung, and liver of *Oasl1* KO mice than in those of WT mice (Fig. 2C). Furthermore, we observed much higher frequencies of virus-specific memory CD8^+^ T cells (CD127^+^) in all the tissues collected from KO mice at 75 d p.i. (Fig. 2D). Even CD127^+^ CD62L^+^ central memory CD8^+^ T cells were easily detectable in the spleen of KO mice at 130 d p.i., but to a lesser extent in WT mice (Fig. 2E). Together, these results indicate that differentiation of virus-specific CD8^+^ T cells progresses more efficiently in *Oasl1* KO mice than in WT mice during the late period of LCMV CL-13 infection.

**Figure 3:**
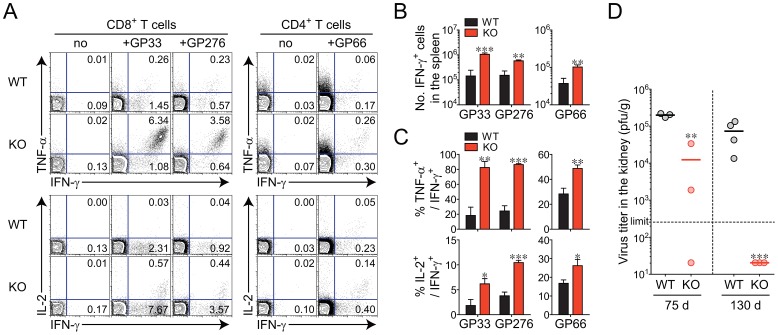
Efficient induction of functional virus-specific T cells and complete viral control in *Oasl1* KO mice during late phase of LCMV infection. (**A**–**C**) Lymphocytes were isolated from the spleens of LCMV CL-13-infected WT and *Oasl1* KO mice at 75 d p.i. and restimulated *in vitro* with GP33 or GP276 peptides for CD8^+^ T cells and GP_66–80_ (GP66) peptide for CD4^+^ T cells. (**A**) Representative data showing the percentages of cytokine-producing cells among CD8^+^ or CD4^+^ T cells. (**B**) Absolute numbers of T cells producing IFN-γ per spleen. (**C**) Summary showing the frequency of TNF-α- or IL-2-producing cells among IFN-γ^+^ CD8^+^ or IFN-γ^+^ CD4^+^ T cells. Bar graphs show mean + SD. (**D**) Virus titers in the kidneys extracted from LCMV CL-13-infected mice at 75 d p.i. (left) and at 130 d p.i. (right). Dashed black line represents the virus detection limit. Undetectable samples were given 20 PFU per gram. The graph includes individual values with their arithmetic mean. All data are representative of at least two independent experiments (*n* = 3–4 per group in each experiment). *, *P*<0.05; **, *P*<0.01; ***, *P*<0.001.

To confirm whether virus-specific T cells in KO mice truly demonstrate better function, we assessed the ability of CD8^+^ and CD4^+^ T cells to produce effector cytokines such as IFN-γ, tumor necrosis factor alpha (TNF-α), and IL-2 at the individual cell level. After *in vitro* restimulation of splenocytes collected at 75 d p.i. with GP_33–41_ or GP_276–286_ peptide for CD8^+^ T cells and GP_66–80_ peptide for CD4^+^ T cells, the IFN-γ-producing T-cell population was detected at a significantly higher level in the *Oasl1* KO sample than in the WT sample (Fig. 3A and 3B). In addition, the percentages of cells producing TNF-α or IL-2 among the IFN-γ-producing cells were much higher in KO mice than in WT mice (Fig. 3A and 3C). The better differentiation and function of virus-specific CD8^+^ T cells observed in *Oasl1* KO mice at 75 d p.i. was maintained at an even later time point (130 d p.i.), although the frequency of LCMV-specific CD8^+^ T cells became similar to that of WT by this time point (Figs. S4 and S5).

Although viral titers in the spleen, lung, and liver of both WT and KO mice at 75 d p.i. were already below a detectable level, the titer in the kidney (a well-known life-long reservoir of chronic LCMV) was detectable, much less in KO mice than in WT mice, indicating better viral control in KO mice (Fig. 3D, data not shown). In addition, at 130 d p.i., *Oasl1* KO mice could eliminate residual viruses completely even in the kidney, whereas WT mice continued to exhibit high viral titers in the kidney (Fig. 3D). These data indicate that the rapid viral control in *Oasl1* KO mice upon LCMV CL-13 infection could be responsible for the enhanced multiple immune responses such as increased virus-specific T-cell function and memory T-cell formation in KO mice, a condition that rarely occurs in WT mice during LCMV CL-13 infection. As a result, *Oasl1* KO mice completely overcame persistent infection at the late stage of infection.

### The major contribution of virus-specific T-cell-extrinsic factors in *Oasl1* KO mice to the expansion of virus-specific CD8^+^ T cells

In the next set of experiments, we attempted to clarify whether the better virus-specific T-cell responses in the absence of OASL1 protein were caused either by virus-specific T-cell-intrinsic change or T-cell-extrinsic change. We also wanted to investigate whether the expanded population of virus-specific CD8^+^ T cells in *Oasl1* KO mice was due to better proliferation or decreased apoptosis of such T cells. To address these questions, we employed LCMV GP_33–41_ epitope-specific T-cell receptor (TCR) transgenic mice (P14 mice). After purifying Thy1.1^+^ CD8^+^ T cells from naïve P14 mice, the P14 Thy1.1^+^ CD8^+^ T cells were adoptively transferred into both naïve WT and *Oasl1* KO mice. The following day, recipient mice were infected with LCMV CL-13 and sacrificed 5 d p.i. when the virus-specific CD8^+^ T-cell population expands enormously.

**Figure 4:**
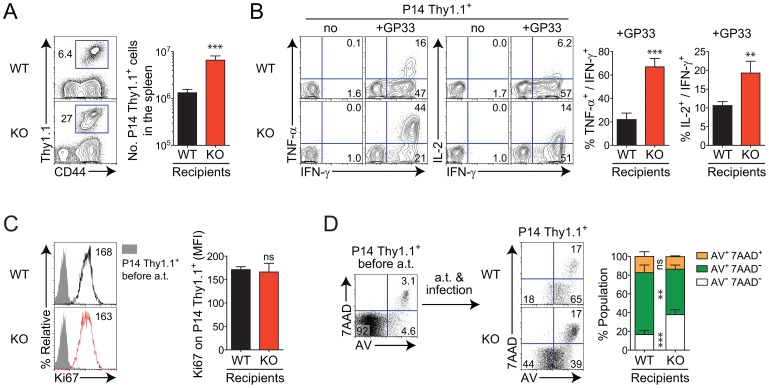
Better responses of virus-specific CD8^+^ T cells and their prolonged survival in *Oasl1* KO mice due to virus-specific T-cell-extrinsic factors. GP33-specific TCR transgenic CD8^+^ T cells, P14 cells, were obtained from naïve P14 Thy1.1 congenic mice. 5×10^3^ of P14 Thy1.1^+^ CD8^+^ T cells were adoptively transferred into WT and *Oasl1* KO mice. The next day, both recipient mice were infected with LCMV CL-13 and splenocytes were isolated from the mice at 5 d p.i. (**A**) Massive expansion of virus-specific donor CD8^+^ T cells in the KO mice. Numbers in the representative plot (left) indicate the frequency of donor P14 Thy1.1^+^ CD8^+^ T cells among splenocytes while the absolute numbers of donor P14 Thy1.1^+^ CD8^+^ T cells present per recipient spleen are summarized in the graph (right). (**B**) Cytokine production from donor P14 Thy1.1^+^ CD8^+^ T cells after *in vitro* restimulation with GP33 peptide. Representative plots (left) are shown for co-expression of IFN-γ and TNF-α or IFN-γ and IL-2 on donor P14 Thy1.1^+^ CD8^+^ T cells; summarized graph (right) for the frequency of TNF-α- or IL-2-producing cells among IFN-γ^+^ CD8^+^ T cells. (**C**) Proliferative capability measurement on donor P14 Thy1.1^+^ CD8^+^ T cells by direct *ex vivo* staining. Representative Ki67 expression levels (MFIs, left) and summarized levels (right) on donor P14 Thy1.1^+^ CD8^+^ T cells are shown. Shade histogram indicates Ki67 expression level on P14 Thy1.1^+^ CD8^+^ T cells before adoptive transfer (a.t.); black line and red line histograms indicate Ki67 expression levels on P14 Thy1.1^+^ CD8^+^ T cells at 5 d p.i. after a.t. into WT and KO mice, respectively. (**D**) Cell death susceptibility of donor P14 Thy1.1^+^ CD8^+^ T cells. Representative (left) and summary (right) data after direct *ex vivo* staining of splenocytes for Annexin V (AV) and 7AAD. Donor P14 Thy1.1^+^ CD8^+^ T cells were analyzed. Data are given as mean + SD. Data are representative of two independent experiments (*n* = 4 per group in each experiment). ns, not significant; **, *P*<0.01; ***, *P*<0.001.

Identical virus-specific P14 CD8^+^ T cells transferred into WT and KO mice displayed noticeably different responses after the chronic virus infection. The P14 donor cells exhibited approximately 3-fold more expansion in *Oasl1* KO mice than in WT mice *in vivo* (Fig. 4A). When restimulated *in vitro* with cognate GP_33–41_ peptide, P14 cells isolated from KO mice at 5 d p.i. generated many more cells co-producing effector cytokines IFN-γ, TNF-α, and IL-2, than those from WT mice (Fig. 4B). These results indicate that virus-specific CD8^+^ T cells respond better to viral antigens in *Oasl1* KO mice both quantitatively and qualitatively. Collectively, these data show that unknown T-cell-extrinsic factors generated in the absence of OASL1 protein play a major role in inciting the donor P14 Thy1.1^+^ CD8^+^ T cells to dramatically increase expansion and cytokine production at the early phase of LCMV CL-13 infection.

When the proliferative capacity of T cells was monitored at 5 d p.i. by measuring the expression of Ki67 (a marker of recently proliferated cells) *ex vivo*, the expression level of Ki67 within the Thy1.1^+^ CD8^+^ T-cell population in *Oasl1* KO mice was similar to that in WT mice (Fig. 4C). However, when the apoptotic phenotype of Thy1.1^+^ CD8^+^ T cells was monitored by measuring the surface expression of Annexin V (AV) and permeability of 7-amino-actinomycin D (7AAD) into cells *ex vivo*, early apoptotic AV^+^ 7AAD^−^ cells were present more frequently in WT mice than in KO mice (Fig. 4D). By contrast, the non-apoptotic population (AV^−^ 7AAD^−^) was much larger in KO mice than in WT mice (Fig. 4D). These results suggest that the extrinsically driven rapid expansion of donor P14 T cells in *Oasl1* KO mice is due to better survival rather than proliferation of donor T cells.

### Prolonged IFN-I production in *Oasl1* KO mice during the early phase of LCMV CL-13 infection as a T-cell-extrinsic factor

**Figure 5:**
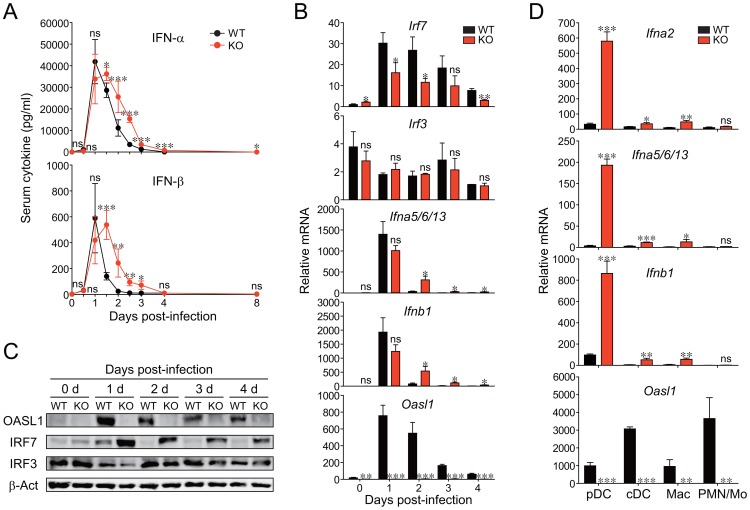
Production of sustained IFN-I and higher IRF7 protein in *Oasl1* KO mice early after LCMV CL-13 infection and the cellular sources of the IFN. WT and *Oasl1* KO mice were infected with LCMV CL-13 and their sera (**A**), spleens (**B–C**), and sorted splenic cells (**D**) were analyzed. (**A**) The levels of IFN-α and IFN-β in the sera were measured by ELISA. Line graphs are shown as mean ± SD. The data are representative of at least four independent experiments (*n*>5 per group in each experiment). (**B**) mRNAs prepared from the spleens were analyzed by quantitative RT-PCR at the indicated days: mRNA expression levels (*n* = 3 per group) normalized to *Gapdh* was recalculated by dividing each expression value with the least mRNA expression value among the samples and shown as a relative mRNA level. (**C**) The protein levels for OASL1, IRF7, IRF3, and β-actin (loading control) were measured by immunoblot. (**D**) mRNA expression levels of IFN-I and OASL1 for the sorted cell populations following the sorting strategy shown in Figure S9. The mRNA expression level (*n* = 3 per group) of *Ifna2*, *Ifna*5*/6/13*, *Ifnb1*, or *Oasl1* normalized to *Gapdh* was shown as a relative mRNA level. All bar graphs show mean + SD. Data of (**B**–**D**) are representative of two independent experiments. ns, not significant; *, *P*<0.05; **, *P*<0.01; ***, *P*<0.001.

To explore which T-cell-extrinsic factors play major roles in the early rapid expansion of CD8^+^ T cells in *Oasl1* KO mice, we measured the levels of major inflammatory cytokines in serum and those of co-stimulatory/MHC molecules expressed on splenic DCs during the early phase of LCMV CL-13 infection. The serum levels of IFN-I were sustained longer in *Oasl1* KO mice than in WT mice from 2 d to 3 d p.i. (Fig. 5A). However, the serum levels of other major cytokines such as IL-6, IL-10, TNF-α, and IFN-γ did not show any meaningful difference between WT and *Oasl1* KO mice at 2 d p.i. but displayed moderately higher signals in KO mice at 3 d p.i. (Fig. S6). This more upregulation of other cytokines at 3 d p.i. in KO mice is thought to be secondarily caused by early sustained IFN-I produced in KO mice [35]–[39]. The expression levels of co-stimulatory molecules and MHC molecules on different subsets of splenic DCs were not higher in *Oasl1* KO mice than in WT mice early after LCMV CL-13 infection (Fig. S7). These results together suggest that IFN-I, a known T-cell survival factor, among extrinsic factors such as cytokines, co-stimulatory molecules, and MHC molecules, sustained longer in *Oasl1* KO mice during the very early stage of infection, may play a critical role in initiating massive expansion of virus-specific CD8^+^ T cells.

### Higher production of IRF7 protein but similar production of IRF3 in LCMV CL-13-infected *Oasl1* KO mice

We previously reported that *Oasl1* KO cells produce more IFN-I upon acute viral infection, which was caused by higher production of IRF7 proteins in KO cells [32]. Thus, we next asked whether chronic LCMV infection also caused higher IRF7 protein production in *Oasl1* KO mice to produce the sustained IFN-I. To this end, we first determined the tissue that had the highest IFN-I expression after LCMV CL-13 infection. Among the four tissues that we collected at 2 d p.i., at the time point when the IFN-I level in serum was clearly stronger in KO mice, the spleen showed the most dominant expression of IFN-I mRNA: a 20-fold more IFN-I mRNA signal compared with other tissues of both WT and KO mice (Fig. S8). Importantly, in the spleen, the IFN-I mRNA level was significantly higher (6- to 8-fold) in KO than in WT mice. Thus, we next focused on spleens to measure the expression of two major TFs responsible for IFN-I mRNA expression during early LCMV CL-13 infection. Upon LCMV CL-13 infection, as expected, IRF7 mRNA (but not IRF3 mRNA), similar to IFN-I mRNA and OASL1 mRNA, was strongly induced at 1 d p.i. and declined over the next several days (Fig. 5B). Similar to serum IFN-I levels (Fig. 5A), *Oasl1* KO spleens expressed much more IFN-I mRNA during the declining several days. Consistent with our previous report that OASL1 specifically inhibits the translation of IRF7 mRNA upon viral infection, IRF7 protein levels were much higher in *Oasl1* KO cells during the days, whereas IRF3 protein levels were similar between WT and KO spleens (Fig. 5C). These results together indicate that the more production of IFN-I in the *Oasl1* KO mice during the early phase of LCMV CL-13 infection was caused by high production of IRF7 proteins in *Oasl1* KO mice.

### Splenic pDCs as a major source of IFN-I during the early phase of LCMV CL-13 infection in *Oasl1* KO mice

We next investigated which cell types are the major source of better IFN-I production in LCMV-CL-13-infected *Oasl1* KO mice. Because major cell types known to produce IFN-I after viral infection *in vivo* are pDCs, conventional DCs (cDCs) including myeloid DCs (mDCs) and lymphoid DCs (lDCs), and Macs [35], we sorted these IFN-I-producing cell types and, as a negative control, polymorphonuclear cells (PMNs) and monocytes (Mos) from splenocytes of WT and *Oasl1* KO mice at 2 d p.i. (Fig. S9), followed by measurement of their IFN-I gene expression levels. Although splenic cDCs and Macs of *Oasl1* KO mice produced more IFN-Is than WT mice, pDCs defined as CD11c^int^B220^+^CD8^+/−^ were the most dominant cellular source for the sustained, higher IFN-I levels observed in the serum of LCMV-CL-13-infected *Oasl1* KO mice at the very early stage of infection: pDCs in *Oasl1* KO mice had more than 10-fold higher IFN-I mRNA levels than cDCs and Macs, and pDCs in *Oasl1* KO mice had a more than 8-fold higher expression of IFN-I mRNAs than those in WT mice (Fig. 5D). However, the expression of OASL1 in these cells was not directly inverse-correlated with IFN-I expression level (Fig. 5D), indicating that other cellular factors also affect IFN-I expression levels (See Discussion).

### The critical role of IFN-I receptor signaling on the better virus-specific CD8^+^ T-cell response in *Oasl1* KO mice

**Figure 6:**
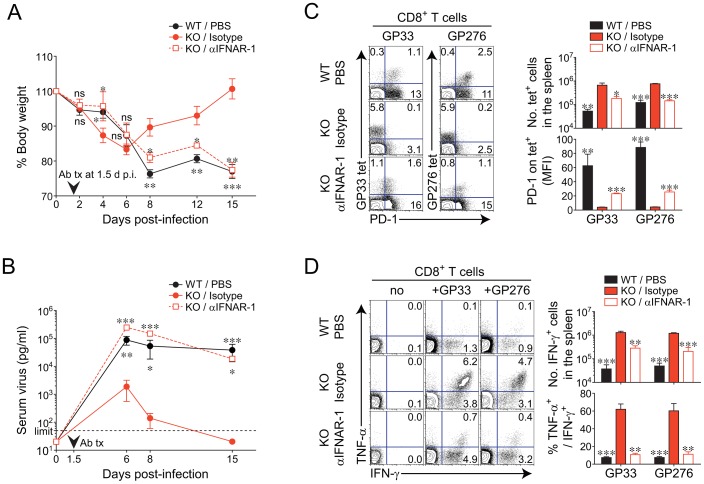
Effect of early *in vivo* blockade of IFN-I receptor signaling on viral clearance and virus-specific CD8^+^ T-cell responses in *Oasl1* KO mice. Mice were infected with LCMV CL-13. At 1.5 d p.i., 0.5 mg IFNAR-1 mAb (αIFNAR-1) or its control isotype Ab (Isotype) was administrated i.v. once into *Oasl1* KO mice. PBS was injected into WT mice as another control. (**A**) Changes in body weight at the indicated time points. (**B**) Serum virus titers of mice at indicated time points p.i. Vertical arrowhead indicates the time point of antibody injection and dashed black line represents the virus detection limit. (**C**) Frequencies of virus (GP33 and GP276)-specific CD8^+^ T cells and their PD-1 expression levels in the spleen at 35 d p.i. Numbers in the plots (left) indicate the percentages of the corresponding cell population among CD8^+^ T cells. Absolute numbers of GP33 tetramer-positive CD8^+^ T cells (top right) and PD-1 MFI values for GP33 tetramer-positive CD8^+^ T cells (bottom right) were also depicted. (**D**) Cytokine production on CD8^+^ T cells obtained from spleens at 35 d p.i. after *in vitro* restimulation with GP33 peptide. Percentages of cytokine-producing cells among CD8^+^ T cells (left) are shown. Absolute numbers of CD8^+^ T cells producing IFN-γ (top right) and the frequencies of TNF-α-producing cells among IFN-γ ^+^ CD8^+^ T cells (bottom right) are also depicted. All line graphs and bar graphs show mean ± SD and mean + SD, respectively. Data are representative of two independent experiments (*n* = 3 per group in each experiment). Statistical significance was determined by comparison between isotype-treated KO mice and each of the other two groups, PBS-treated WT mice or αIFNAR-1-treated KO mice. ns, not significant; *, *P*<0.05; **, *P*<0.01; ***, *P*<0.001.

We next asked whether IFN-I receptor signaling in *Oasl1* KO mice is essential for the massive expansion of CD8^+^ T cells and the accelerated viral control in KO mice. To address this question, we performed *in vivo* blockade experiments using an antibody against the IFN-α/β receptor 1 (IFNAR-1), which efficiently blocks IFN-I receptor signaling caused by IFN-I [40], [41]. At 1.5 d after LCMV CL-13 infection, the time point just before the serum level of IFN-I began to show a real significant difference between WT and *Oasl1* KO mice (Fig. 5A), *Oasl1* KO mice were treated with either IFNAR-1 antibody or its isotype control, and WT mice were treated with phosphate-buffered saline (PBS) as another control. IFNAR-1 antibody-treated *Oasl1* KO mice and PBS-treated WT mice displayed much more similar patterns in both body weight changes and serum viremia than isotype-control-treated *Oasl1* KO mice (Figs. 6A and 6B). Furthermore, the spleen and blood of IFNAR-1 antibody-treated *Oasl1* KO mice showed rather typical phenotypes of CD8^+^ T cells that have been observed in chronically-infected WT mice, such as a moderate frequency of LCMV-specific CD8^+^ T cells, high expression of PD-1, and impaired induction of effector cytokines (Fig. 6C and 6D). By contrast, isotype control-treated *Oasl1* KO mice did not show such chronic infection phenotypes. Collectively, these data indicate that the better virus-specific CD8^+^ T-cell responses and accelerated virus control in *Oasl1* KO mice need IFN-I receptor signaling triggered by sustained IFN-I in KO mice.

## Discussion

In the present study, we showed that *Oasl1* KO mice produced sustained level of IFN-I during the very early phase (2–3 d p.i.) of chronic LCMV CL-13 infection, cleared the virus quickly (earlier than 15 d p.i.) from the blood circulation, and induced rapid expansion of virus-specific effector T cells. We also showed that *Oasl1* KO mice cleared the virus even from the kidney, a place for long-lasting existence of LCMV CL-13, at the late phase of infection (by 130 d p.i.) and induced the production of polyfunctional virus-specific memory CD8^+^ T cells. Furthermore, we showed that IFN-I receptor signaling during the very early stage of LCMV CL-13 infection was necessary for the higher T-cell number, efficient T-cell differentiation, and early viral control in *Oasl1* KO mice.

The sustained production of IFN-I and more effective viral clearance in *Oasl1* KO mice after chronic LCMV CL-13 infection observed in the present study resembles our previous observation that *Oasl1* KO mice are more resistant to acute infections with encephalomyocarditis virus and herpes simplex virus-1 and produce higher levels of IFN-I [32]. Because OASL1 is a specific translation inhibitor of IRF7, the IFN-inducible IFN-I master TF, the sustained and stronger expression of IFN-I in *Oasl1* KO mice upon LCMV infection is thought to be caused by the release of such inhibition and effective activation of the more translated IRF7 protein in KO mice. Indeed, we observed that the IRF7 protein level in *Oasl1* KO mice was much higher during the early phase of chronic LCMV CL-13 infection (Fig. 5C). Because most viruses induce IFN-I and in turn IRF7 expression and can activate the resulting IRF7 protein effectively, *Oasl1* KO mice are expected to be resistant to most viral infections that do not produce viral effectors to degrade the function of IRF7. Consistent with this expectation, *Oasl1* KO mice turned out to be more resistant to chronic LCMV CL-13 infection as well as acute LCMV Arm infection (Figs. 1F and S3), although LCMV is equipped with nucleoprotein (NP), a virus-derived IFN-I negative regulator that interferes with activation of IRF3 [42]–[44]. In both acute LCMV Arm and chronic LCMV CL-13 infections, OASL1 expression was similarly strongly induced (>20-fold) during the very early phase of infection, and then declined gradually (1–4 d p.i.) (Fig. S10). However, at later time points (9–15 d p.i.), only with LCMV CL-13 infection, OASL1 expression was still marginally up-regulated (Fig. S10), which would reflect the presence of viruses in the mice infected with LCMV CL-13 (Figs. 1F and S3). Therefore, as expected, the mRNA expression of OASL1 (as an ISG) is dominantly regulated by IFN-I induced upon viral infection.

The cellular source of IFN-I upon chronic LCMV CL-13 infection seems to include pDCs and other innate immune cell types such as cDCs and Macs as recently reported [45], [46]. Among these cells, we showed in our study that the critical cellular source for the sustained and higher serum IFN-I level in *Oasl1* KO mice at the early stage of infection (2 d p.i. when the IFN-I level in serum became clearly stronger in the KO mice than in WT mice) was pDCs (CD11c^int^B220^+^CD8^+/−^), although cDCs and Macs of *Oasl1* KO mice also produced significantly more IFN-I than those of WT mice (Fig. 5D). However, IFN-I expression in these cells was not inversely correlated with OASL1 expression in these cells, indicating that other factors also affect the expression of IFN-I. The pDC-dominant IFN-I production in *Oasl1* KO mice at the early stage of LCMV CL-13 infection could be explained by the differences in expressed viral sensors and in IRF7 expression levels among these cells, with the demonstrated role of OASL1 as a specific IRF7 translation inhibitor. It has been well known that cDCs and Macs express viral sensors such as TLR3 and RIG-I/MDA5 that sense dsRNA produced during viral replication, including that of the ssRNA virus LCMV, whereas pDCs dominantly express TLR7 that sense ssRNAs and TLR9 that sense DNAs among known viral sensors [35], [47]. Because the IRF7 expression level in pDCs is much higher than that in cDCs and Macs at a steady state (data not shown) [48], [49], and cell membrane-associated receptor TLR7 can sense the ssRNA viral genome directly even without viral replication upon LCMV infection, pDCs are expected to produce IFN-I initially by sensing the viral genome first and inducing activation of IRF7 protein [4], [45]. After initial production of IFN-I, pDCs would amplify IFN-I production by inducing an IFN-I-mediated positive feedback signaling loop in an autocrine fashion, leading also to accumulation of IRF7 mRNA [4], [50]. In this later phase of IFN-I production, IRF7 protein levels would be much higher in *Oasl1* KO pDCs than in WT pDCs because OASL1 protein derived from *Oasl1* mRNA induced by the IFN-I-mediated positive feedback signaling loop would inhibit IRF7 translation in WT cells. Thus, *Oasl1* KO pDCs could produce much higher levels of IFN-I at the very early stage of LCMV infection: we observed a more than 8-fold higher IFN-I level in KO cells than in WT cells (Fig. 5D).

In addition to reducing viral titers during the early phase of viral infection, such a sustained level of IFN-I in *Oasl1* KO mice might act directly and indirectly on CD8^+^ T cells to enhance their numbers and function. IFN-I receptor signaling in CD8^+^ T cells triggered by the sustained IFN-I level in KO mice appears to be critical for the massive expansion of virus-specific CD8^+^ T cells, given that such signaling in CD8^+^ T cells is known to prevent apoptosis of T cells *in vivo*, allowing clonal expansion and differentiation upon viral infection [51]–[56]. CD4^+^ T cells could also be a direct target for IFN-I in KO mice because IFN-I was reported to act directly on CD4^+^ T cells to sustain their clonal expansion [57] and promote Th1 differentiation *in vivo* [58]–[60]. In addition to the direct IFN-I effect on T cells, IFN-I was reported to enhance the function of DCs by inducing maturation, such as upregulation of costimulatory molecules and MHC class molecules [61]–[63]. However, a sustained and increased level of IFN-I in *Oasl1* KO mice does not seem to critically affect DC maturation in the setting of chronic LCMV infection because there was no apparent difference in the expression of costimulatory molecules and MHCs in DCs isolated from WT and *Oasl1* KO mice (Fig. S7). The level of IFN-I produced in WT mice could be sufficient to induce DC maturation, and its higher and sustained level in *Oasl1* KO mice might not create any additional effects on DC maturation. It would be interesting, in the future, to determine whether other types of cells influenced by IFN-I also contribute to the better virus-specific CD8^+^ T-cell response observed in *Oasl1* KO mice.

The complete elimination of chronic virus and efficient induction of the adaptive immune response in *Oasl1* KO mice observed in this study is extraordinary because WT mice infected with chronic LCMV CL-13 suffer from a sustained viral load throughout their life spans and have fundamentally dysfunctional T cells [34]. There has been considerable number of molecules known to significantly affect viral persistence like an OASL1. For example, functional blockage of IL-10, a well-known immunosuppressive molecule, during the chronic phase of LCMV CL-13 infection reduced viral load; IL-21 receptor signaling in CD8^+^ T cells was critical for limiting LCMV viral load *in vivo*; IL-7, the major T-cell survival factor, enhanced LCMV viral clearance; the functional blockage of PD-1, a T-cell-intrinsic signaling inhibitor, led to LCMV viral load reduction [19], [64]–[66]. However, most of these previously characterized molecules, if not all, are not related directly to IFN-I production and its regulation but are mostly involved in controlling adaptive immune responses. Therefore, our report demonstrating the critical role of OASL1, as a direct negative regulator of IFN-I production, in permitting chronic viral persistence is, to our knowledge, the first demonstration of a role of any host IFN-I negative regulator in viral clearance in the setting of chronic infection.

The negative role of OASL1, as an IFN-I negative regulator, in viral clearance suggests that inhibition of the expression/function of OASL1 or IFN-I treatment during the early phase of chronic viral infections could prevent viral persistence. Indeed, a recent study showed that IFN-I treatment in WT mice at 3 d and 5 d p.i. (when the IFN-I level is downregulated after its initial peak) after LCMV CL-13 infection dramatically reduced the LCMV viral load and promoted virus-specific CD8 T-cell responses [46]. This observation is consistent with our data that a sustained level of IFN-I at approximately 2–4 d p.i. in *Oasl1* KO mice led to rapid control of viremia and functional virus-specific T-cell responses. However, in the previous study, IFN-I treatment in WT mice later than 1 week of infection did not have any meaningful effect on viral load [46], suggesting that the timing of IFN-I treatment is critical for controlling viremia and restoring the T-cell response and that other factors may degrade IFN-I-mediated responses. Given that the ineffectiveness of exogenous IFN-I at the later phase of chronic virus infection, it is worthwhile to note that *Oasl1* expression in pDCs was still sustained at a later phase (30 d p.i.) of chronic LCMV CL-13 infection but not of acute LCMV Arm infection, whereas *Ifna/b1* expression in the pDCs returned to the basal level by that phase in both LCMV infections (Fig. S11). Therefore, it is possible that, at the later phase of chronic viral infection, exogenously administered IFN-I itself is not sufficient to induce an adequate level of IFN-I production for viral clearance because the production of IRF7 protein, the key player necessary for going through the IFN-I-mediated positive feedback signaling loop, would still be inhibited by OASL1 protein. In this regard, suppression of OASL1 function or expression during the chronic phase of LCMV infection may help to eliminate the virus by releasing OASL1-mediated suppression of IFN-I production, although we cannot exclude the possibility that other factors, including other IFN-I negative regulators, still promote viral persistence.

Our results showing that reversal of the defective production of IFN-I during chronic virus infection by nullifying OASL1 can inhibit viral persistence raise the possibility that inhibiting the functions of other negative regulators acting on the process of IFN-I production and/or IFN-I receptor signaling pathways would improve the host defense against chronic viral infections. Peptidyl-prolyl cis/trans isomerase NIMA-interacting 1 (Pin 1), which induces degradation of IRF3 protein, and two ubiquitin E3 ligases, tripartite motif-containing 21 (TRIM21) and RTA-associated ubiquitin ligase (RAUL), which induce the degradation of both IRF3 and IRF7 proteins [28], [67], are host factors more directly involved in negatively regulating IFN-I production. Negative host factors involved in IFN-I receptor signaling pathways include inhibitors of the JAK-STAT signaling pathway downstream of the IFN-I receptor such as Suppressor of cytokine signaling (SOCS), Src homology 2-containing protein tyrosine phosphatase (SHP), and protein inhibitors of activated STAT (PIAS) [68], [69]. Because most of the currently known negative factors not only regulate IFN-I but also other cytokines [28], OASL1 is a rather unique host negative factor regulating IFN-I production that specifically functions in IRF7 protein production. In the future, a combinatorial approach for controlling the expression and/or function of OASL1 and regulating diverse other targets might be worthy of investigation. However, blockade of virus-encoded IFN-I suppressors may also be necessary for these approaches to be more effective.

## Materials and Methods

### Ethics statement

All animal experiments were performed in accordance with the Korean Food and Drug Administration (KFDA) guidelines. Protocols were reviewed and approved by the Institutional Animal Care and Use Committee (IACUC) of the Yonsei Laboratory Animal Research Center (YLARC) at Yonsei University (Permit Number: 2007-0001).

### Mice, infection, and titration

C57BL/6 mice were purchased from the Jackson Laboratory, and LCMV CL-13 epitope-specific TCR transgenic P14 Thy1.1 mice were obtained from the Emory Vaccine Center. *Oasl1* KO mice were derived from our previous study [32]. All mice were maintained in the specific pathogen-free facility of the YLARC at Yonsei University. Six- to ten-week-old littermate female or male C57BL/6 WT and *Oasl1* KO mice were infected with 2×10^6^ plaque-forming units (PFUs) of LCMV CL-13 or LCMV Arm diluted in serum-free RPMI medium per 20 g of mouse body weight by intravenous (i.v.) injection. For serum virus titration, three to five drops of blood were collected at the indicated time points p.i., and the serum was directly stored at −70°C. For tissue virus titration, small pieces of the spleen, lung, liver, and kidney, were put in Dulbecco's modified Eagle's medium (DMEM) containing 1% fetal bovine serum (FBS) (Gibco) and stored at −70°C. The tissues were later homogenized completely using a homogenizer (Kinematica) before titration. Viral titers from sera or homogenized samples were determined by plaque assay on Vero cells as previously described [33].

### Cell isolation, antibodies, and staining

PBMCs were isolated from peripheral blood using density gradient centrifugation underlaid with histopaque-1077 (Sigma-Aldrich). Lymphocytes from tissues, including the spleen, lung, and liver, were isolated as previously described [9]. Lungs and livers were perfused with ice-cold PBS before collection for lymphocyte isolation. For phenotypic analysis of virus-specific CD8^+^ T cells derived from peripheral blood and tissues, single-cell suspensions were stained with CD4 (RM4-5), CD8a (53-6.7), CD44 (IM7), CD62L (MEL-14), CD127 (A7R34), and PD-1 (RMP1-30) antibodies in the presence of each tetramer. H-2D^b^ tetramers bound to GP_33–41_ and GP_276–286_ peptides were generated and used as previously described [70]. Pieces of spleen were digested with 1 mg/ml of type II collagenase (Worthington Biochemicals) and 1 mg/ml of bovine pancreatic DNase I (Sigma-Aldrich) as previously described for the analysis of splenic DCs, Macs, and PMNs/Mos at the indicated time points after LCMV CL-13 infection [71]. The resulting splenocytes were mixed with CD16/CD32 (2.4G2) antibodies and stained with CD3e (145-2C11), CD19 (1D3), CD49b (DX5), CD11c (HL-3), CD11b (M1/70), F4/80 (BM8), CD8a (53-6.7), and B220 (RA3-6B2) antibodies. Splenocytes were co-stained with CD40 (3/23), CD80 (16-10A1), CD86 (GL1), H-2K^b^ (AF6-88.5), I-A/I-E (M5/114.15.2), and PD-L1 (MIH5) antibodies, or their isotype antibodies to detect various accessory molecules in different subset of DCs. After 5 h of *in vitro* restimulation of splenocytes with 0.2 µg/ml of LCMV GP_33–41_ or GP_276–286_ peptide to induce a CD8^+^ T-cell response, and with 1.0 µg/ml of GP_66–80_ peptide to induce a CD4^+^ T-cell response, in the presence of Golgi plug/Golgi stop (BD Biosciences), intracellular staining for cytokines was performed using IFN-γ (XMG1.2), TNF-α (MP6-XT22), and IL-2 (JES6-5H4) antibodies. To measure the proliferation capability *ex vivo*, splenocytes were stained with a Ki67 antibody (B56). For direct analysis of apoptosis *ex vivo*, splenocytes were briefly incubated with Annexin V and 7AAD (BD Biosciences). All antibodies were purchased from BD Biosciences except for CD127, CD11b, F4/80, I-A/I-E, PD-L1 (eBioscience), and PD-1 antibodies (BioLegend). The Live/Dead fixable dead cell Stain kit (Invitrogen) was used to remove the dead cell population in most staining procedures, except in *ex vivo* apoptosis staining. All stained samples were read using FACSCalibur and FACSCantoII instruments (BD Biosciences) and analyzed using FlowJo software (Tree Star).

### Cell sorting and adoptive transfer

The splenocytes digested with type II collagenase and DNase I were incubated with a cocktail of biotin-conjugated antibodies and anti-biotin beads (Miltenyi Biotech) and were subsequently depleted of T, B, and natural killer (NK) cells. The resulting splenocytes were mixed with CD16/CD32 (2.4G2) antibody and incubated with CD11c beads (Miltenyi Biotech). After magnetic separation, the bead-attached cells were stained with CD11c (HL-3), CD8a (53-6.7), and B220 (RA3-6B2) antibodies for sorting cDCs and pDCs, and the unattached cells were stained with CD11b (M1/70) and F4/80 (BM8) antibodies for sorting Macs and PMNs/Mos. The stained cells were sorted using a FACSAriaII instrument (BD Biosciences). For adoptive transfer of P14 Thy1.1^+^ CD8^+^ T cells, cells were isolated from the spleen of B6 P14 Thy1.1^+^ mice using a CD8^+^ T-cell isolation kit (Miltenyi Biotec). Mice were infected with LCMV CL-13 at 1 d after the adoptive transfer of 5×10^3^ P14 Thy1.1^+^ CD8^+^ T cells via the tail vein into naïve WT or *Oasl1* KO mice.

### Enzyme-linked immunosorbent assay (ELISA), cytometric bead array (CBA), and immunoblot analysis

Sera were obtained from WT and KO mice at the indicated time points after LCMV CL-13 infection for measurement of cytokines. The IFN-I levels in the sera were measured using VeriKine Mouse Interferon Alpha and Beta ELISA kits (PBL interferon source). Other cytokine levels in the sera were measured using BD CBA mouse inflammation kits (BD Biosciences) according to the manufacturer's instructions. For immunoblot analysis, snap-frozen tissues were homogenized in NP-40 buffer [50 mM Tris-HCl (pH 8.0), 150 mM NaCl, and 1% NP-40] with a protease inhibitor ‘cocktail’ (1 mM PMSF, 10 µg/ml aprotinin, 5 µg/ml pepstatin and 5 µg/ml leupeptin) by sonication, and the tissue lysate was centrifugated at 17,000× *g* for 10 min at 4°C for removal of tissue debris. Equal amounts of proteins were analyzed by immunoblotting using IRF7 (51-3300, Zymed), IRF3 (51-3200, Zymed), β-actin (A2228, Sigma-Aldrich) and OASL1 [32] antibodies, and signals developed with Amersham ECL reagents were detected using the ImageQuant LAS 4000 system (GE Healthcare).

### *In vivo* blockade of IFN-I receptor signaling

To block IFN-I receptor signaling *in vivo*, 0.5 mg (per mouse) of anti-mouse IFNAR-1 monoclonal antibody (clone MAR1-5A3; BD Biosciences) was administered to *Oasl1* KO mice 1.5 d p.i. by i.v. injection. PBS and mouse IgG1 (MOPC-21, isotype control) were injected into WT and KO mice, respectively, at the same time point. Body weight was monitored every 2, 3, or 4 d, and serum was collected at 6, 8, and 15 d p.i. All mice were sacrificed at 35 d p.i., and their spleens were harvested and analyzed.

### RNA analysis by quantitative reverse transcription-polymerase chain reaction (RT-PCR)

Tissues from animals were snap-frozen in liquid nitrogen after perfusion with 5 mM ethylenediaminetetraacetic acid in PBS and sorted cells were pelleted by centrifugation before lysis. Total RNA from the tissues and cells were purified using TRIzol RNA Isolation Reagent (Invitrogen), and cDNAs were synthesized using SuperScript II Reverse Transcriptase according to the manufacturer's protocol (Invitrogen). The expression levels of individual genes were measured by quantitative PCR (qPCR) performed using the CFX96 real-time PCR detection system (Bio-Rad) with the following gene-specific forward (F) and reverse (R) primers: *Oasl1* F: CCAGGAAGAAGCCAAGCACCATC and R: AGGTTACTGAGCCCAAGGTCCATC; *Irf7* F: CAGCAGCAGTCTCGGCTTGTG and R: TGACCCAGGTCCATGAGGAAGTG; *Irf3* F: CTGGACGAGAGCCGAACGAG and R: TGTAGGCACCACTGGCTTCTG; *Ifna2* F: AAGGACAGGCAGGACTTTGGATTC and R: GATCTCGCAGCACAGGGATGG; *Ifna5/6/13* F: AGGACTCATCTGCTGCATGGAATG and R: CACACAGGCTTTGAGGTCATTGAG; *Ifnb1* F: CCACTTGAAGAGCTATTACTG and R: AATGATGAGAAAGTTCCTGAAG; *Gapdh* F: GGCAAATTCAACGGCACAGTCAAG and R: TCGCTCCTGGAAGATGGTGATGG. The relative mRNA expression normalized to the *Gapdh* signal was shown. The qPCR reaction conditions were as follows: after initial denaturation of the template for 3 min at 95°C, 45 thermal cycles of 15 sec at 95°C, 25 sec at 60°C, and 30 sec at 72°C were run in a final volume of 20 µl using SYBR Green I dye for PCR product detection (Qiagen) according to the manufacturer's instructions.

### Statistical analysis

Statistical analysis of all presented data was performed using a two-tailed unpaired Student's *t*-test using Prism 5.0 software (GraphPad). A *P* value less than 0.05 was considered statistically significant.

### Sequence information for genes and proteins

The list of GenBank accession numbers for the major genes and proteins that are mentioned in the text are as follows: OASL1, NP_660210 (NM_145209); TLR3, NP_569054 (NM_126166); TLR7, NP_573474 (NM_133211); TLR9, NP_112455 (NM_031178); RIG-I, NP_766277 (NM_172689); MDA5, NP_001157949 (NM_001164477)); IFN-α, NP_034632 (NM_010502); IFN-β, NP_034640 (NM_010510); PD-1, NP_032824 (NM_008798); TIM-3, NP_599011 (NM_134250); CTLA-4, NP_033973 (NM_009843); LAG-3, NP_032505 (NM_008479); IRF3, NP_058545 (NM_016849); IRF7, NP_058546 (NM_016850); Pin 1, NP_075860 (NM_023371); TRIM21, NP_001076021 (NM_001082552); RAUL, NP_001003918 (NM_001003918); IFNAR, NP_034638 (NM_010508); PIAS, NP_001159421 (NM_001165949).

## Supporting Information

Figure S1 **No significant upregulation of PD-1 but fast recovery of CD127 expression on virus-specific CD8^+^ T cells of PBMCs in** ***Oasl1*** **KO mice after LCMV CL-13 infection.** WT and *Oasl1* KO mice were infected with LCMV CL-13 and PBMCs were collected at indicated time points p.i. (A) Representative data showing PD-1 expression levels on both GP33 tetramer-positive and -negative CD8^+^ T cells (left panel) and its levels on GP33 tetramer-positive CD8^+^ T cells (right panel). (B) Representative data showing CD127 expression levels on both GP33 tetramer-positive and negative CD8^+^ T cells (left panel) and its levels on GP33 tetramer-positive CD8^+^ T cells (right panel). Blue vertical lines in the plots of (B) divide CD127^+^ and CD127^−^ tetramer-positive CD8^+^ T cells. Numbers in contour plots of (A) and (B) indicate the percentages for the corresponding cell populations. Numbers in histograms represent the value of PD-1 MFI (A) and the percentage of CD127^+^ cells among GP33 tetramer-positive CD8^+^ T cells (B). Data are representative of four independent experiments (*n*>6 per group in each experiment).
(PDF)

Figure S2 **Rapid recovery of the body weight in** ***Oasl1*** **KO mice during LCMV CL-13 infection.** WT and *Oasl1* KO mice were infected with LCMV CL-13, and their body weights were monitored at the indicated times. Data are representative of four independent experiments (*n*>6 per group in each experiment). ns, not significant; ***, *P*<0.001.
(PDF)

Figure S3 **Serum virus titers in WT and** ***Oasl1*** **KO mice post-infection with LCMV Arm or CL-13 infection.** WT and *Oasl1* KO mice were infected intravenously with 2×10^6^ pfu of LCMV Arm or CL-13 via tail vein and virus titers were measured in the serum at the indicated time points p.i. Data are represented as mean + SD (*n* = 3–6 mice per group). ns, not significant; *, *P*<0.05; ***, *P*<0.001.
(PDF)

Figure S4 **Frequency and phenotype of virus-specific CD8^+^ T cells in tissues of** ***Oasl1*** **KO mice at a late time point after LCMV CL-13 infection.** Lymphocytes were isolated from the spleen (SP), lung (LG), and liver (LV) of LCMV CL-13-infected WT and *Oasl1* KO mice at 130 d p.i. (A) Representative data showing the frequency of GP33 and GP276 tetramer-positive cells among CD8^+^ T cells. (B) Absolute numbers of tetramer-positive CD8^+^ T cells in the indicated tissues. (C, D) PD-1 and CD127 expression levels on LCMV-specific CD8^+^ T cells in the spleen of WT and KO mice. Numbers in the plots (left) indicate PD-1 MFI value (C) for GP33 and GP276 tetramer-positive CD8^+^ T cells and CD127^+^ cell percentage (D) among the tetramer-positive cells. Blue vertical lines in the plots of (D) divide CD127^+^ and CD127^−^ tetramer-positive CD8^+^ T cells. The PD-1 MFI values and CD127^+^ cell frequency in the indicated tissues are also summarized in bar graphs as mean + SD (right). Data are representative of at least two independent experiments (*n* = 3–4 per group in each experiment). ns, not significant; *, *P*<0.05; **, *P*<0.01; ***, *P*<0.001.
(PDF)

Figure S5 **Better induction of functional virus-specific CD8^+^ T cells in** ***Oasl1*** **KO mice at a late time point after LCMV CL-13 infection.** Lymphocytes were isolated from the spleen of LCMV CL-13-infected WT and *Oasl1* KO mice at 130 d p.i. and restimulated *in vitro* with GP33 or GP276 peptides. (A) Representative data for cytokine production on CD8^+^ T cells. Percentages of cytokine-producing cells among CD8^+^ T cells are shown in the plots. (B) Absolute numbers of CD8^+^ T cells producing IFN-γ in the spleen. (C) Summary showing the frequency of TNF-α- or IL-2-producing cells among IFN-γ^+^ CD8^+^ T cells. All bar graphs show mean + SD. All data are representative of at least two independent experiments (*n* = 3–4 per group in each experiment). ns, not significant; *, *P*<0.05; **, *P*<0.01; ***, *P*<0.001.
(PDF)

Figure S6 **Production patterns of major inflammatory cytokines early after LCMV CL-13 infection.** Sera were collected from LCMV-infected WT and *Oasl1* KO mice at the indicated time points, and the levels of six major mouse inflammatory cytokines were measured by cytometric bead array assay. IL-12 level was below a detectable limit. All bar graphs display mean + SD. Data are representative of two independent experiments (*n*>5 per group in each experiment). ns, not significant; *, *P*<0.05; **, *P*<0.01; ***, *P*<0.001.
(PDF)

Figure S7 **Expressions of major costimulatory and MHC class molecules on different DC subsets of WT and** ***Oasl1*** **KO mice at early time points after LCMV CL-13 infection.** Splenocytes were isolated from uninfected (0 d) and LCMV CL-13-infected WT and *Oasl1* KO mice at the indicated time points p.i. The DC populations were defined as CD3^−^CD19^−^CD49b^−^CD11c^+^ cells and further gated on myeloid DCs (mDC, CD8^−^B220^−^), lymphoid DCs (lDC, CD8^+^B220^−^), and plasmacytoid DCs (pDC, CD8^+/−^B220^+^). Expressions of various costimulatory molecules, MHC class molecules, and PD-L1 on different subsets of DCs are depicted with their MFI values and shown as mean ± SD. Data are representative of two independent experiments (*n* = 3–4 per group in each experiment).
(PDF)

Figure S8 **Dominant expression of IFN-I mRNAs in the spleen of LCMV CL-13-infected mice at 2 d p.i.** WT and *Oasl1* KO mice were infected with LCMV CL-13 and the spleen (SP), lung (LG), liver (LV), and kidney (KD) were collected at 2 d p.i. *Ifna2* and *Ifnb1* mRNA expression levels normalized to *Gapdh* mRNA amount were recalculated by dividing each expression value with the least mRNA expression value among the samples and shown as a relative mRNA level. Data are shown as mean + SD. Three mice were used for each group. ns, not significant; *, *P*<0.05; ***, *P*<0.001.
(PDF)

Figure S9 **Splenic cell sorting strategy and post-sort verification of purity.** (A) To obtain homogenous populations for splenic conventional DCs (cDC) and plasmacytoid DCs (pDC) (left panel), and for splenic macrophages (Mac) and polymorphonuclear cells/monocytes (PMN/Mo) (right panel), the splenocytes from LCMV CL-13-infected WT and *Oasl1* KO mice (pooled from at least 10 mice per group) were isolated at 2 d p.i. and sorted by using flow cytometry. The purified cells were then re-analyzed for purity check. (B) CD11c expression level of the sorted pDC (black line) and cDC (red line) from WT and *Oasl1* KO mice. Numbers in the plot indicate MFI value of CD11c expressed by pDC (black letter) and cDC (red letter).
(PDF)

Figure S10 **Comparison of** ***Oasl1*** **mRNA expression levels in the spleen after infection with LCMV Arm or CL-13.** WT mice were uninfected (0 d) or infected with the same dosage (2×10^6^ PFU/mice) of LCMV Arm or CL-13 and via the same route and their spleens were collected at the indicated time points p.i. *Oasl1* mRNA expression level normalized to *Gapdh* mRNA amount was recalculated by dividing each expression value with the least mRNA expression value among the samples. Data are representative of two independent experiments (*n* = 3 per group in each experiment) and shown as mean + SD. ns, not significant; *, *P*<0.05; **, *P*<0.01.
(PDF)

Figure S11 **Gene expression levels of** ***Oasl1*** **and** ***Ifn-I*** **on pDCs at 1 and 30 d p.i.** WT B6 mice were infected i.v. with 2×10^6^ PFU of LCMV Arm or CL-13. Spleens were obtained from naive (0 d p.i.) and virus-infected mice 1 d and 30 d p.i. Single cell-suspended splenocytes were depleted of T cells, B cells, NK cells, and granulocytes by bead depletion. After enrichment of splenic CD11c^+^ cells using CD11c beads, the cells stained with anti-CD11c, CD8a, and B220 Abs were sorted to obtain plasmacytoid DCs using the strategy shown in Figure S9. (A) Virus titers in the spleens. At each time point, spleens obtained from naïve, Arm-, and CL-13-infected mice were homogenized to measure virus titers. Dashed black line represents the virus detection limit. (B, C) Relative mRNA expression levels of *Oasl1* (n = 3 per group) (B) and *Ifn-I* (C) including *Ifna2*, *Ifna5*, and *Ifnb1* on pDC at 0, 1, and 30 d p.i. All bar graphs show mean + SD. ns, not significant; *, *P*<0.05; ***, *P*<0.001.
(PDF)

## References

[1] Lee MS, Kim YJ (2007) Signaling pathways downstream of pattern-recognition receptors and their cross talk. Annu Rev Biochem 76: 447–480. 17328678 10.1146/annurev.biochem.76.060605.122847

[2] Takeuchi O, Akira S (2009) Innate immunity to virus infection. Immunol Rev 227: 75–86. 19120477 10.1111/j.1600-065X.2008.00737.x PMC5489343

[3] Barbalat R, Ewald SE, Mouchess ML, Barton GM (2011) Nucleic acid recognition by the innate immune system. Annu Rev Immunol 29: 185–214. 21219183 10.1146/annurev-immunol-031210-101340

[4] Kawai T, Akira S (2006) Innate immune recognition of viral infection. Nat Immunol 7: 131–137. 16424890 10.1038/ni1303

[5] Iwasaki A, Medzhitov R (2004) Toll-like receptor control of the adaptive immune responses. Nat Immunol 5: 987–995. 15454922 10.1038/ni1112

[6] Iwasaki A, Medzhitov R (2010) Regulation of adaptive immunity by the innate immune system. Science 327: 291–295. 20075244 10.1126/science.1183021 PMC3645875

[7] Virgin HW, Wherry EJ, Ahmed R (2009) Redefining chronic viral infection. Cell 138: 30–50. 19596234 10.1016/j.cell.2009.06.036

[8] West EE, Youngblood B, Tan WG, Jin HT, Araki K, et al (2011) Tight regulation of memory CD8(+) T cells limits their effectiveness during sustained high viral load. Immunity 35: 285–298. 21856186 10.1016/j.immuni.2011.05.017 PMC3241982

[9] Barber DL, Wherry EJ, Masopust D, Zhu B, Allison JP, et al (2006) Restoring function in exhausted CD8 T cells during chronic viral infection. Nature 439: 682–687. 16382236 10.1038/nature04444

[10] Petrovas C, Casazza JP, Brenchley JM, Price DA, Gostick E, et al (2006) PD-1 is a regulator of virus-specific CD8+ T cell survival in HIV infection. J Exp Med 203: 2281–2292. 16954372 10.1084/jem.20061496 PMC2118095

[11] Jin HT, Anderson AC, Tan WG, West EE, Ha SJ, et al (2010) Cooperation of Tim-3 and PD-1 in CD8 T-cell exhaustion during chronic viral infection. Proc Natl Acad Sci U S A 107: 14733–14738. 20679213 10.1073/pnas.1009731107 PMC2930455

[12] Kaufmann DE, Kavanagh DG, Pereyra F, Zaunders JJ, Mackey EW, et al (2007) Upregulation of CTLA-4 by HIV-specific CD4+ T cells correlates with disease progression and defines a reversible immune dysfunction. Nat Immunol 8: 1246–1254. 17906628 10.1038/ni1515

[13] Blackburn SD, Shin H, Haining WN, Zou T, Workman CJ, et al (2009) Coregulation of CD8+ T cell exhaustion by multiple inhibitory receptors during chronic viral infection. Nat Immunol 10: 29–37. 19043418 10.1038/ni.1679 PMC2605166

[14] Jin HT, Jeong YH, Park HJ, Ha SJ (2011) Mechanism of T cell exhaustion in a chronic environment. BMB Rep 44: 217–231. 21524346 10.5483/BMBRep.2011.44.4.217

[15] Abbas AK, Sharpe AH (2005) Dendritic cells giveth and taketh away. Nat Immunol 6: 227–228. 15716967 10.1038/ni0305-227

[16] Ebinuma H, Nakamoto N, Li Y, Price DA, Gostick E, et al (2008) Identification and in vitro expansion of functional antigen-specific CD25+ FoxP3+ regulatory T cells in hepatitis C virus infection. J Virol 82: 5043–5053. 18337568 10.1128/JVI.01548-07 PMC2346728

[17] Vignali DA, Collison LW, Workman CJ (2008) How regulatory T cells work. Nat Rev Immunol 8: 523–532. 18566595 10.1038/nri2343 PMC2665249

[18] Brooks DG, Trifilo MJ, Edelmann KH, Teyton L, McGavern DB, et al (2006) Interleukin-10 determines viral clearance or persistence in vivo. Nat Med 12: 1301–1309. 17041596 10.1038/nm1492 PMC2535582

[19] Ejrnaes M, Filippi CM, Martinic MM, Ling EM, Togher LM, et al (2006) Resolution of a chronic viral infection after interleukin-10 receptor blockade. J Exp Med 203: 2461–2472. 17030951 10.1084/jem.20061462 PMC2118120

[20] Alatrakchi N, Graham CS, van der Vliet HJ, Sherman KE, Exley MA, et al (2007) Hepatitis C virus (HCV)-specific CD8+ cells produce transforming growth factor beta that can suppress HCV-specific T-cell responses. J Virol 81: 5882–5892. 17376924 10.1128/JVI.02202-06 PMC1900307

[21] Li MO, Sanjabi S, Flavell RA (2006) Transforming growth factor-beta controls development, homeostasis, and tolerance of T cells by regulatory T cell-dependent and -independent mechanisms. Immunity 25: 455–471. 16973386 10.1016/j.immuni.2006.07.011

[22] Kanto T, Inoue M, Miyatake H, Sato A, Sakakibara M, et al (2004) Reduced numbers and impaired ability of myeloid and plasmacytoid dendritic cells to polarize T helper cells in chronic hepatitis C virus infection. J Infect Dis 190: 1919–1926. 15529255 10.1086/425425

[23] Duan XZ, Wang M, Li HW, Zhuang H, Xu D, et al (2004) Decreased frequency and function of circulating plasmocytoid dendritic cells (pDC) in hepatitis B virus infected humans. J Clin Immunol 24: 637–646. 15622448 10.1007/s10875-004-6249-y

[24] Finke JS, Shodell M, Shah K, Siegal FP, Steinman RM (2004) Dendritic cell numbers in the blood of HIV-1 infected patients before and after changes in antiretroviral therapy. J Clin Immunol 24: 647–652. 15622449 10.1007/s10875-004-6250-5

[25] Altfeld M, Fadda L, Frleta D, Bhardwaj N (2011) DCs and NK cells: critical effectors in the immune response to HIV-1. Nat Rev Immunol 11: 176–186. 21350578 10.1038/nri2935 PMC3278081

[26] Zuniga EI, Liou LY, Mack L, Mendoza M, Oldstone MB (2008) Persistent virus infection inhibits type I interferon production by plasmacytoid dendritic cells to facilitate opportunistic infections. Cell Host Microbe 4: 374–386. 18854241 10.1016/j.chom.2008.08.016 PMC2875928

[27] Lee LN, Burke S, Montoya M, Borrow P (2009) Multiple mechanisms contribute to impairment of type 1 interferon production during chronic lymphocytic choriomeningitis virus infection of mice. J Immunol 182: 7178–7189. 19454715 10.4049/jimmunol.0802526

[28] Richards KH, Macdonald A (2011) Putting the brakes on the anti-viral response: negative regulators of type I interferon (IFN) production. Microbes Infect 13: 291–302. 21256242 10.1016/j.micinf.2010.12.007

[29] Bowie AG, Unterholzner L (2008) Viral evasion and subversion of pattern-recognition receptor signalling. Nat Rev Immunol 8: 911–922. 18989317 10.1038/nri2436 PMC7097711

[30] Akhtar LN, Benveniste EN (2011) Viral exploitation of host SOCS protein functions. J Virol 85: 1912–1921. 21084484 10.1128/JVI.01857-10 PMC3067810

[31] Aghemo A, Rumi MG, Colombo M (2010) Pegylated interferons alpha2a and alpha2b in the treatment of chronic hepatitis C. Nat Rev Gastroenterol Hepatol 7: 485–494. 20644567 10.1038/nrgastro.2010.101

[32] Lee MS, Kim B, Oh GT, Kim YJ (2013) OASL1 inhibits translation of the type I interferon-regulating transcription factor IRF7. Nat Immunol 14: 346–355. 23416614 10.1038/ni.2535

[33] Wherry EJ, Blattman JN, Murali-Krishna K, van der Most R, Ahmed R (2003) Viral persistence alters CD8 T-cell immunodominance and tissue distribution and results in distinct stages of functional impairment. J Virol 77: 4911–4927. 12663797 10.1128/JVI.77.8.4911-4927.2003 PMC152117

[34] Wherry EJ, Ahmed R (2004) Memory CD8 T-cell differentiation during viral infection. J Virol 78: 5535–5545. 15140950 10.1128/JVI.78.11.5535-5545.2004 PMC415833

[35] Gonzalez-Navajas JM, Lee J, David M, Raz E (2012) Immunomodulatory functions of type I interferons. Nat Rev Immunol 12: 125–135. 22222875 10.1038/nri3133 PMC3727154

[36] Chang EY, Guo B, Doyle SE, Cheng G (2007) Cutting edge: involvement of the type I IFN production and signaling pathway in lipopolysaccharide-induced IL-10 production. J Immunol 178: 6705–6709. 17513714 10.4049/jimmunol.178.11.6705

[37] Mancuso G, Midiri A, Biondo C, Beninati C, Zummo S, et al (2007) Type I IFN signaling is crucial for host resistance against different species of pathogenic bacteria. J Immunol 178: 3126–3133. 17312160 10.4049/jimmunol.178.5.3126

[38] Nguyen KB, Watford WT, Salomon R, Hofmann SR, Pien GC, et al (2002) Critical role for STAT4 activation by type 1 interferons in the interferon-gamma response to viral infection. Science 297: 2063–2066. 12242445 10.1126/science.1074900

[39] Zhu J, Huang X, Yang Y (2007) Innate immune response to adenoviral vectors is mediated by both Toll-like receptor-dependent and -independent pathways. J Virol 81: 3170–3180. 17229689 10.1128/JVI.02192-06 PMC1866082

[40] Dunn GP, Bruce AT, Sheehan KC, Shankaran V, Uppaluri R, et al (2005) A critical function for type I interferons in cancer immunoediting. Nat Immunol 6: 722–729. 15951814 10.1038/ni1213

[41] Sheehan KC, Lai KS, Dunn GP, Bruce AT, Diamond MS, et al (2006) Blocking monoclonal antibodies specific for mouse IFN-alpha/beta receptor subunit 1 (IFNAR-1) from mice immunized by in vivo hydrodynamic transfection. J Interferon Cytokine Res 26: 804–819. 17115899 10.1089/jir.2006.26.804

[42] Martinez-Sobrido L, Emonet S, Giannakas P, Cubitt B, Garcia-Sastre A, et al (2009) Identification of amino acid residues critical for the anti-interferon activity of the nucleoprotein of the prototypic arenavirus lymphocytic choriomeningitis virus. J Virol 83: 11330–11340. 19710144 10.1128/JVI.00763-09 PMC2772779

[43] Martinez-Sobrido L, Giannakas P, Cubitt B, Garcia-Sastre A, de la Torre JC (2007) Differential inhibition of type I interferon induction by arenavirus nucleoproteins. J Virol 81: 12696–12703. 17804508 10.1128/JVI.00882-07 PMC2168988

[44] Martinez-Sobrido L, Zuniga EI, Rosario D, Garcia-Sastre A, de la Torre JC (2006) Inhibition of the type I interferon response by the nucleoprotein of the prototypic arenavirus lymphocytic choriomeningitis virus. J Virol 80: 9192–9199. 16940530 10.1128/JVI.00555-06 PMC1563941

[45] Macal M, Lewis GM, Kunz S, Flavell R, Harker JA, et al (2012) Plasmacytoid Dendritic Cells Are Productively Infected and Activated through TLR-7 Early after Arenavirus Infection. Cell Host Microbe 11: 617–630. 22704622 10.1016/j.chom.2012.04.017 PMC3377983

[46] Wang Y, Swiecki M, Cella M, Alber G, Schreiber RD, et al (2012) Timing and Magnitude of Type I Interferon Responses by Distinct Sensors Impact CD8 T Cell Exhaustion and Chronic Viral Infection. Cell Host Microbe 11: 631–642. 22704623 10.1016/j.chom.2012.05.003 PMC3572910

[47] Kawai T, Akira S (2010) The role of pattern-recognition receptors in innate immunity: update on Toll-like receptors. Nat Immunol 11: 373–384. 20404851 10.1038/ni.1863

[48] Dai J, Megjugorac NJ, Amrute SB, Fitzgerald-Bocarsly P (2004) Regulation of IFN regulatory factor-7 and IFN-alpha production by enveloped virus and lipopolysaccharide in human plasmacytoid dendritic cells. J Immunol 173: 1535–1548. 15265881 10.4049/jimmunol.173.3.1535

[49] Izaguirre A, Barnes BJ, Amrute S, Yeow WS, Megjugorac N, et al (2003) Comparative analysis of IRF and IFN-alpha expression in human plasmacytoid and monocyte-derived dendritic cells. J Leukoc Biol 74: 1125–1138. 12960254 10.1189/jlb.0603255

[50] Honda K, Takaoka A, Taniguchi T (2006) Type I interferon [corrected] gene induction by the interferon regulatory factor family of transcription factors. Immunity 25: 349–360. 16979567 10.1016/j.immuni.2006.08.009

[51] Curtsinger JM, Valenzuela JO, Agarwal P, Lins D, Mescher MF (2005) Type I IFNs provide a third signal to CD8 T cells to stimulate clonal expansion and differentiation. J Immunol 174: 4465–4469. 15814665 10.4049/jimmunol.174.8.4465

[52] Kolumam GA, Thomas S, Thompson LJ, Sprent J, Murali-Krishna K (2005) Type I interferons act directly on CD8 T cells to allow clonal expansion and memory formation in response to viral infection. J Exp Med 202: 637–650. 16129706 10.1084/jem.20050821 PMC2212878

[53] Thompson LJ, Kolumam GA, Thomas S, Murali-Krishna K (2006) Innate inflammatory signals induced by various pathogens differentially dictate the IFN-I dependence of CD8 T cells for clonal expansion and memory formation. J Immunol 177: 1746–1754. 16849484 10.4049/jimmunol.177.3.1746

[54] Cervantes-Barragan L, Lewis KL, Firner S, Thiel V, Hugues S, et al (2012) Plasmacytoid dendritic cells control T-cell response to chronic viral infection. Proc Natl Acad Sci U S A 109: 3012–3017. 22315415 10.1073/pnas.1117359109 PMC3286988

[55] Wiesel M, Kratky W, Oxenius A (2011) Type I IFN substitutes for T cell help during viral infections. J Immunol 186: 754–763. 21160039 10.4049/jimmunol.1003166

[56] Pinto AK, Daffis S, Brien JD, Gainey MD, Yokoyama WM, et al (2011) A temporal role of type I interferon signaling in CD8+ T cell maturation during acute West Nile virus infection. PLoS Pathog 7: e1002407. 22144897 10.1371/journal.ppat.1002407 PMC3228803

[57] Havenar-Daughton C, Kolumam GA, Murali-Krishna K (2006) Cutting Edge: The direct action of type I IFN on CD4 T cells is critical for sustaining clonal expansion in response to a viral but not a bacterial infection. J Immunol 176: 3315–3319. 16517698 10.4049/jimmunol.176.6.3315

[58] Huber JP, Farrar JD (2011) Regulation of effector and memory T-cell functions by type I interferon. Immunology 132: 466–474. 21320124 10.1111/j.1365-2567.2011.03412.x PMC3075500

[59] Matikainen S, Paananen A, Miettinen M, Kurimoto M, Timonen T, et al (2001) IFN-alpha and IL-18 synergistically enhance IFN-gamma production in human NK cells: differential regulation of Stat4 activation and IFN-gamma gene expression by IFN-alpha and IL-12. Eur J Immunol 31: 2236–2245. 11449378

[60] Strengell M, Julkunen I, Matikainen S (2004) IFN-alpha regulates IL-21 and IL-21R expression in human NK and T cells. J Leukoc Biol 76: 416–422. 15178704 10.1189/jlb.1003488

[61] Gallucci S, Lolkema M, Matzinger P (1999) Natural adjuvants: endogenous activators of dendritic cells. Nat Med 5: 1249–1255. 10545990 10.1038/15200

[62] Luft T, Pang KC, Thomas E, Hertzog P, Hart DN, et al (1998) Type I IFNs enhance the terminal differentiation of dendritic cells. J Immunol 161: 1947–1953. 9712065

[63] Montoya M, Schiavoni G, Mattei F, Gresser I, Belardelli F, et al (2002) Type I interferons produced by dendritic cells promote their phenotypic and functional activation. Blood 99: 3263–3271. 11964292 10.1182/blood.v99.9.3263

[64] Elsaesser H, Sauer K, Brooks DG (2009) IL-21 is required to control chronic viral infection. Science 324: 1569–1572. 19423777 10.1126/science.1174182 PMC2830017

[65] Frohlich A, Kisielow J, Schmitz I, Freigang S, Shamshiev AT, et al (2009) IL-21R on T cells is critical for sustained functionality and control of chronic viral infection. Science 324: 1576–1580. 19478140 10.1126/science.1172815

[66] Pellegrini M, Calzascia T, Toe JG, Preston SP, Lin AE, et al (2011) IL-7 engages multiple mechanisms to overcome chronic viral infection and limit organ pathology. Cell 144: 601–613. 21295337 10.1016/j.cell.2011.01.011

[67] Yu Y, Hayward GS (2010) The ubiquitin E3 ligase RAUL negatively regulates type i interferon through ubiquitination of the transcription factors IRF7 and IRF3. Immunity 33: 863–877. 21167755 10.1016/j.immuni.2010.11.027 PMC3012379

[68] Naka T, Fujimoto M, Tsutsui H, Yoshimura A (2005) Negative regulation of cytokine and TLR signalings by SOCS and others. Adv Immunol 87: 61–122. 16102572 10.1016/S0065-2776(05)87003-8

[69] Shuai K, Liu B (2003) Regulation of JAK-STAT signalling in the immune system. Nat Rev Immunol 3: 900–911. 14668806 10.1038/nri1226

[70] Murali-Krishna K, Altman JD, Suresh M, Sourdive DJ, Zajac AJ, et al (1998) Counting antigen-specific CD8 T cells: a reevaluation of bystander activation during viral infection. Immunity 8: 177–187. 9491999 10.1016/s1074-7613(00)80470-7

[71] Ha SJ, Mueller SN, Wherry EJ, Barber DL, Aubert RD, et al (2008) Enhancing therapeutic vaccination by blocking PD-1-mediated inhibitory signals during chronic infection. J Exp Med 205: 543–555. 18332181 10.1084/jem.20071949 PMC2275378

