# Supplementary figures and images for: Negative Regulation of Type I IFN Expression by OASL1 Permits Chronic Viral Infection and CD8+ T-Cell Exhaustion
Source: PLoS Pathog. 2013 Jul 18;9(7):e1003478. doi: 10.1371/journal.ppat.1003478 (PMC3715418; doi:10.1371/journal.ppat.1003478)

A

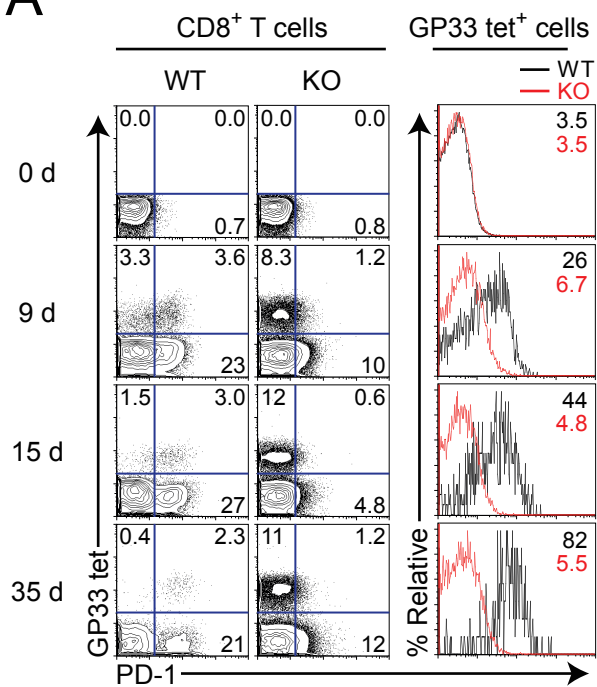

B

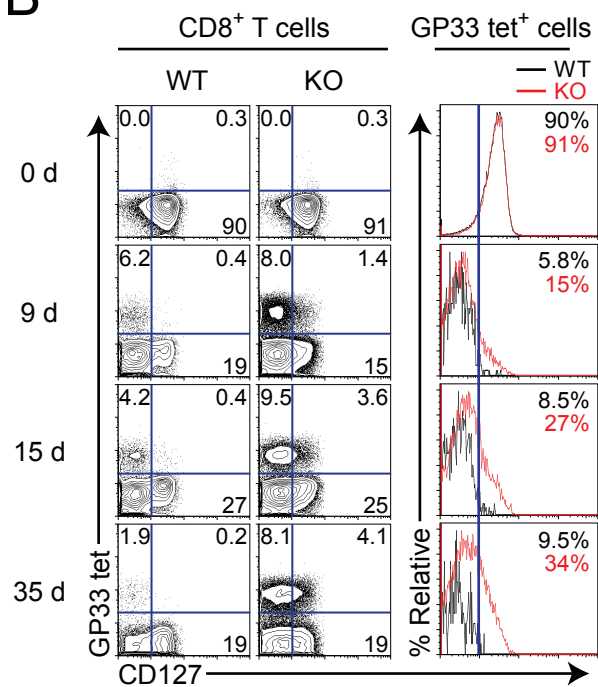

Supplement: Figure S1 — No significant upregulation of PD-1 but fast recovery of CD127 expression on virus-specific CD8+ T cells of PBMCs in Oasl1 KO mice after LCMV CL-13 infection. WT and Oasl1 KO mice were infected with LCMV CL-13 and PBMCs were collected at indicated time points p.i. (A) Representative data showing PD-1 expression levels on both GP33 tetramer-positive and -negative CD8+ T cells (left panel) and its levels on GP33 tetramer-positive CD8+ T cells (right panel). (B) Representative data showing CD127 expression levels on both GP33 tetramer-positive and negative CD8+ T cells (left panel) and its levels on GP33 tetramer-positive CD8+ T cells (right panel). Blue vertical lines in the plots of (B) divide CD127+ and CD127− tetramer-positive CD8+ T cells. Numbers in contour plots of (A) and (B) indicate the percentages for the corresponding cell populations. Numbers in histograms represent the value of PD-1 MFI (A) and the percentage of CD127+ cells among GP33 tetramer-positive CD8+ T cells (B). Data are representative of four independent experiments (n>6 per group in each experiment). (PDF) [file ppat.1003478.s001.pdf]

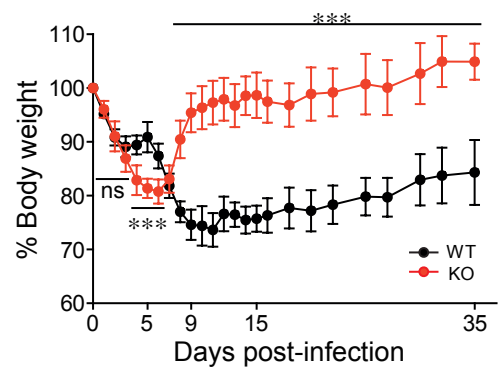

Supplement: Figure S2 — Rapid recovery of the body weight in Oasl1 KO mice during LCMV CL-13 infection. WT and Oasl1 KO mice were infected with LCMV CL-13, and their body weights were monitored at the indicated times. Data are representative of four independent experiments (n>6 per group in each experiment). ns, not significant; ***, P<0.001. (PDF) [file ppat.1003478.s002.pdf]

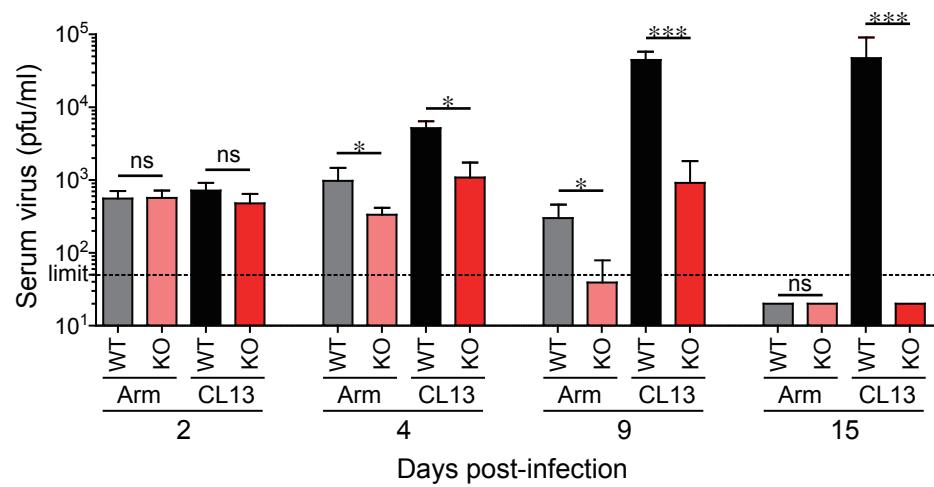

Supplement: Figure S3 — Serum virus titers in WT and Oasl1 KO mice post-infection with LCMV Arm or CL-13 infection. WT and Oasl1 KO mice were infected intravenously with 2×10^6^ pfu of LCMV Arm or CL-13 via tail vein and virus titers were measured in the serum at the indicated time points p.i. Data are represented as mean + SD (n = 3–6 mice per group). ns, not significant; *, P<0.05; ***, P<0.001. (PDF) [file ppat.1003478.s003.pdf]

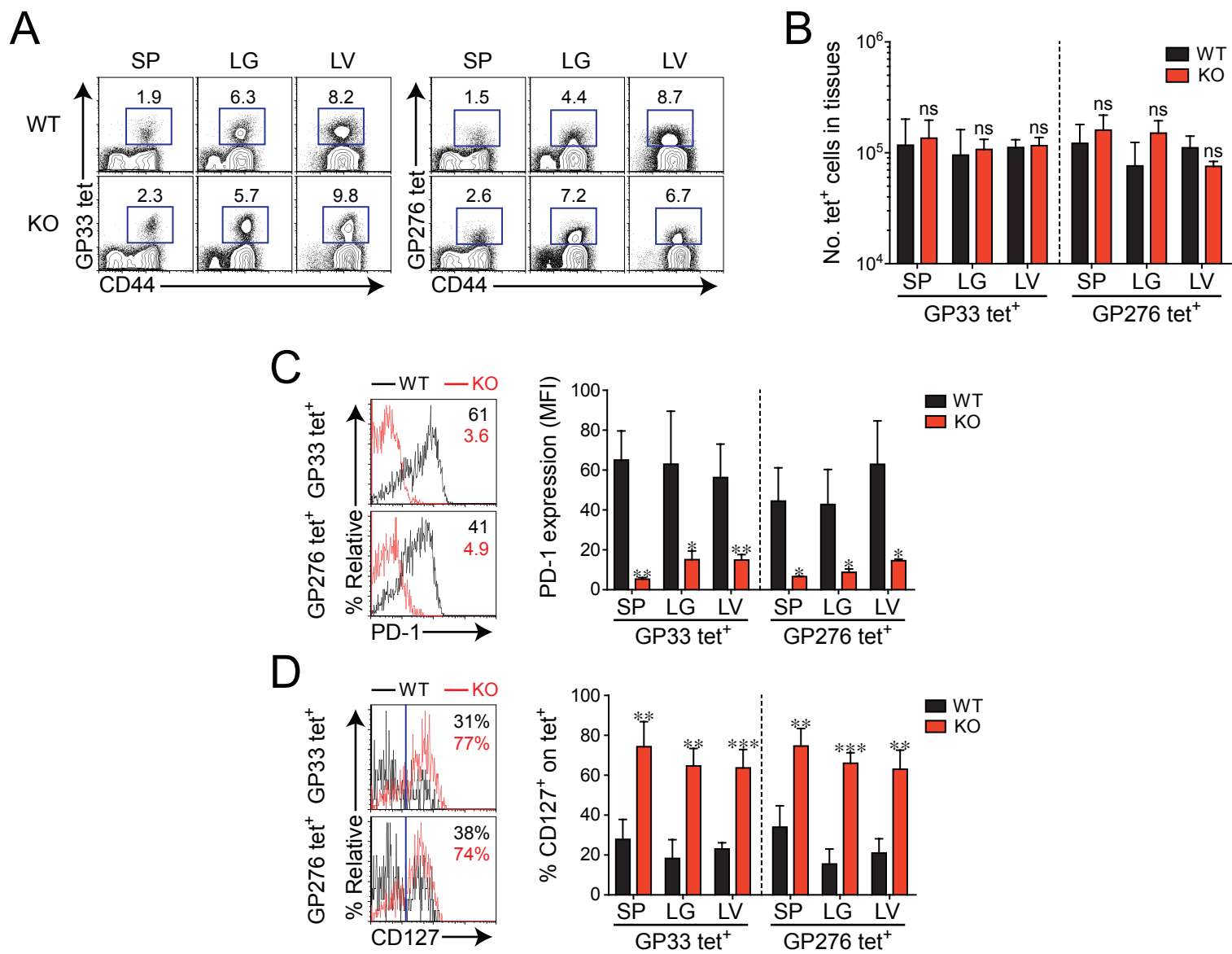

Supplement: Figure S4 — Frequency and phenotype of virus-specific CD8+ T cells in tissues of Oasl1 KO mice at a late time point after LCMV CL-13 infection. Lymphocytes were isolated from the spleen (SP), lung (LG), and liver (LV) of LCMV CL-13-infected WT and Oasl1 KO mice at 130 d p.i. (A) Representative data showing the frequency of GP33 and GP276 tetramer-positive cells among CD8+ T cells. (B) Absolute numbers of tetramer-positive CD8+ T cells in the indicated tissues. (C, D) PD-1 and CD127 expression levels on LCMV-specific CD8+ T cells in the spleen of WT and KO mice. Numbers in the plots (left) indicate PD-1 MFI value (C) for GP33 and GP276 tetramer-positive CD8+ T cells and CD127+ cell percentage (D) among the tetramer-positive cells. Blue vertical lines in the plots of (D) divide CD127+ and CD127− tetramer-positive CD8+ T cells. The PD-1 MFI values and CD127+ cell frequency in the indicated tissues are also summarized in bar graphs as mean + SD (right). Data are representative of at least two independent experiments (n = 3–4 per group in each experiment). ns, not significant; *, P<0.05; **, P<0.01; ***, P<0.001. (PDF) [file ppat.1003478.s004.pdf]

A

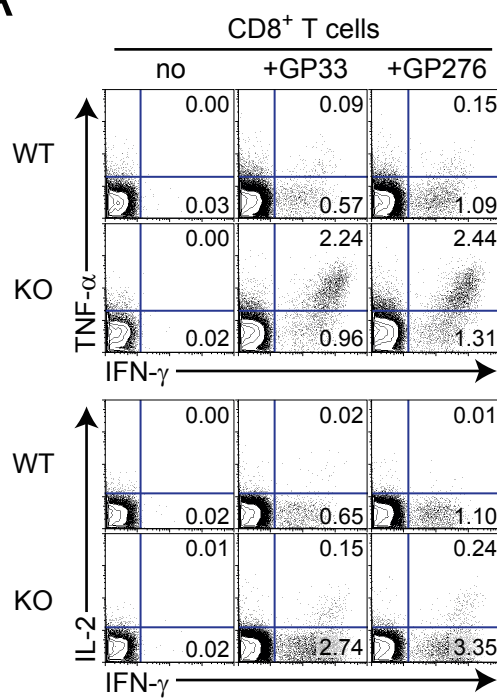

B

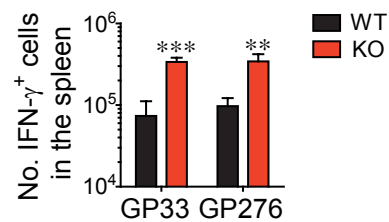

C

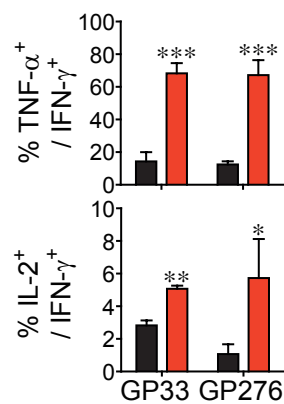

Supplement: Figure S5 — Better induction of functional virus-specific CD8+ T cells in Oasl1 KO mice at a late time point after LCMV CL-13 infection. Lymphocytes were isolated from the spleen of LCMV CL-13-infected WT and Oasl1 KO mice at 130 d p.i. and restimulated in vitro with GP33 or GP276 peptides. (A) Representative data for cytokine production on CD8+ T cells. Percentages of cytokine-producing cells among CD8+ T cells are shown in the plots. (B) Absolute numbers of CD8+ T cells producing IFN-γ in the spleen. (C) Summary showing the frequency of TNF-α- or IL-2-producing cells among IFN-γ+ CD8+ T cells. All bar graphs show mean + SD. All data are representative of at least two independent experiments (n = 3–4 per group in each experiment). ns, not significant; *, P<0.05; **, P<0.01; ***, P<0.001. (PDF) [file ppat.1003478.s005.pdf]

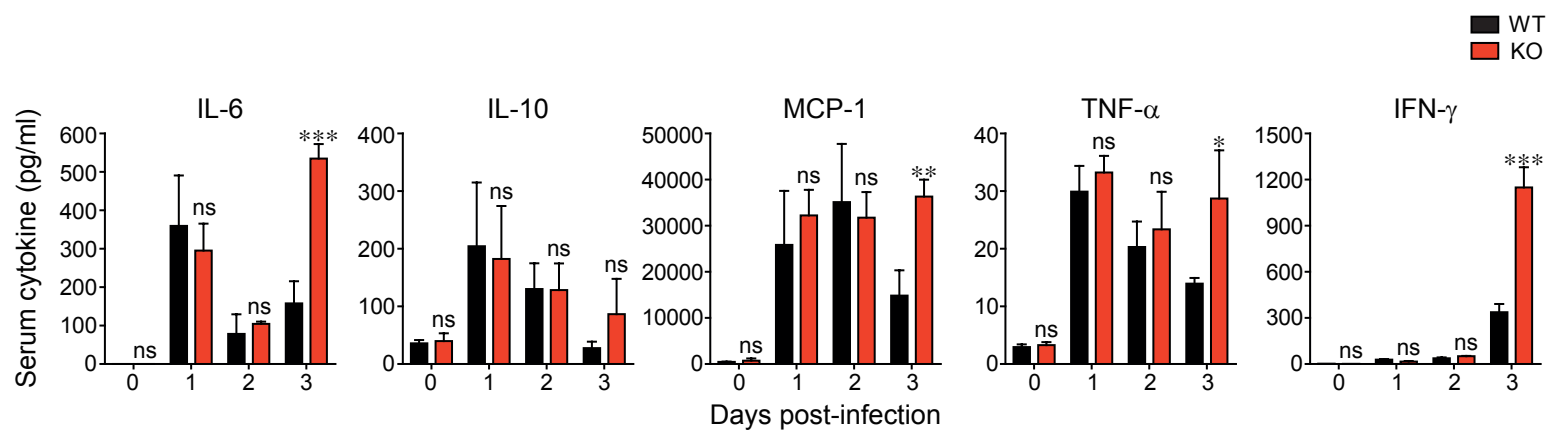

Supplement: Figure S6 — Production patterns of major inflammatory cytokines early after LCMV CL-13 infection. Sera were collected from LCMV-infected WT and Oasl1 KO mice at the indicated time points, and the levels of six major mouse inflammatory cytokines were measured by cytometric bead array assay. IL-12 level was below a detectable limit. All bar graphs display mean + SD. Data are representative of two independent experiments (n>5 per group in each experiment). ns, not significant; *, P<0.05; **, P<0.01; ***, P<0.001. (PDF) [file ppat.1003478.s006.pdf]

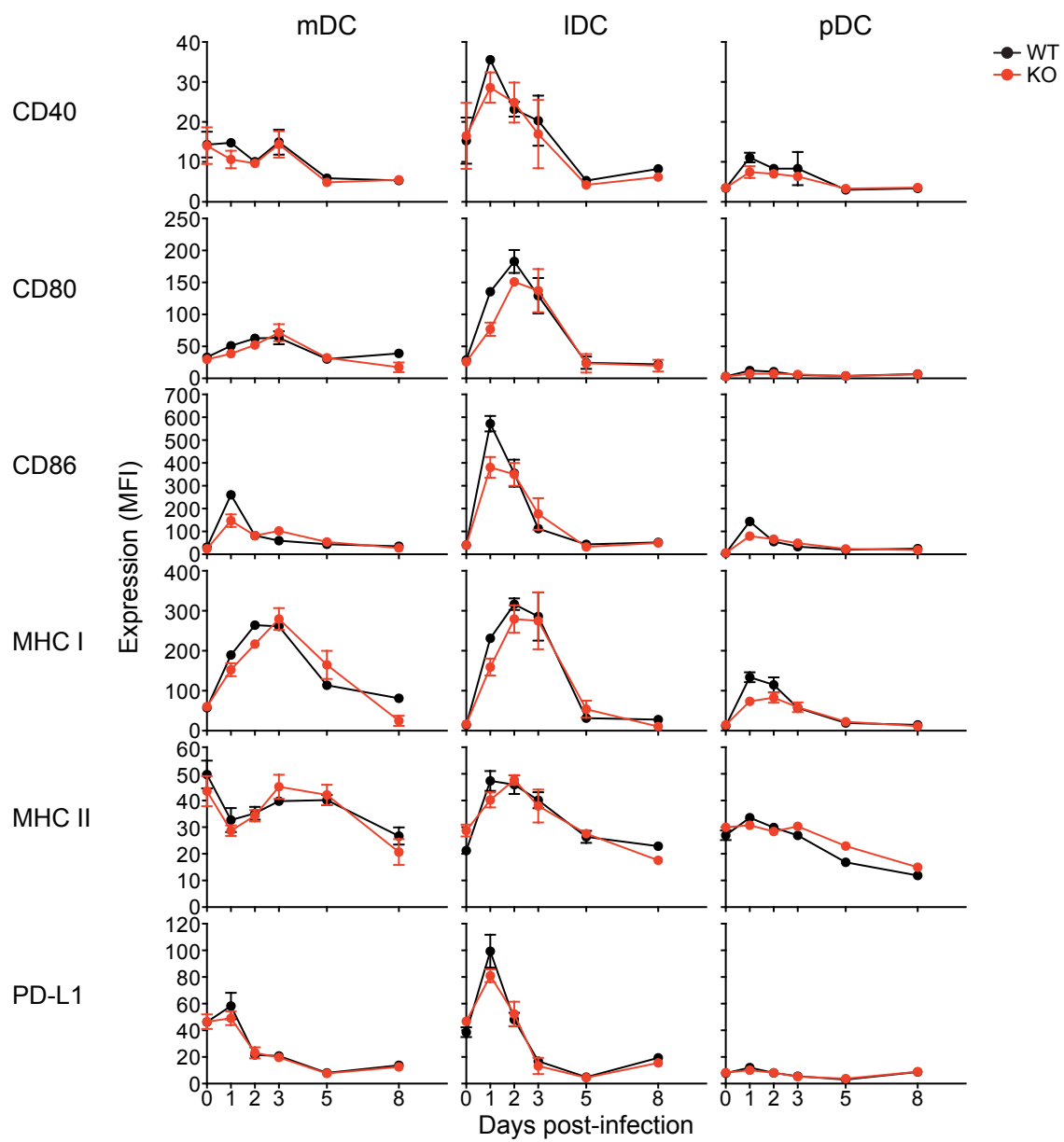

Supplement: Figure S7 — Expressions of major costimulatory and MHC class molecules on different DC subsets of WT and Oasl1 KO mice at early time points after LCMV CL-13 infection. Splenocytes were isolated from uninfected (0 d) and LCMV CL-13-infected WT and Oasl1 KO mice at the indicated time points p.i. The DC populations were defined as CD3−CD19−CD49b−CD11c+ cells and further gated on myeloid DCs (mDC, CD8−B220−), lymphoid DCs (lDC, CD8+B220−), and plasmacytoid DCs (pDC, CD8+/−B220+). Expressions of various costimulatory molecules, MHC class molecules, and PD-L1 on different subsets of DCs are depicted with their MFI values and shown as mean ± SD. Data are representative of two independent experiments (n = 3–4 per group in each experiment). (PDF) [file ppat.1003478.s007.pdf]

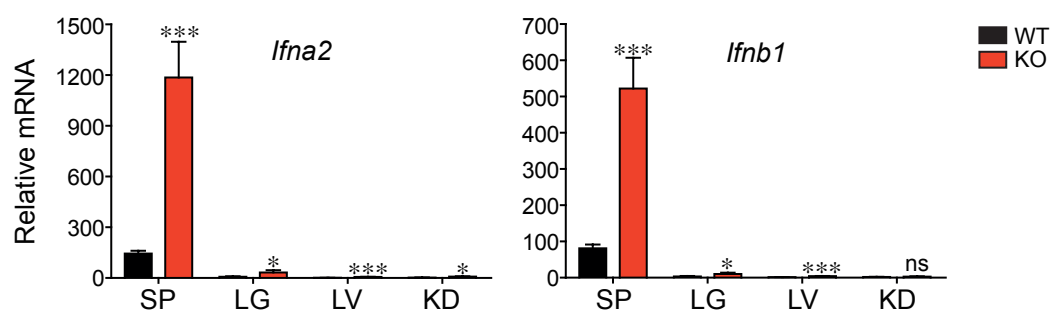

Supplement: Figure S8 — Dominant expression of IFN-I mRNAs in the spleen of LCMV CL-13-infected mice at 2 d p.i. WT and Oasl1 KO mice were infected with LCMV CL-13 and the spleen (SP), lung (LG), liver (LV), and kidney (KD) were collected at 2 d p.i. Ifna2 and Ifnb1 mRNA expression levels normalized to Gapdh mRNA amount were recalculated by dividing each expression value with the least mRNA expression value among the samples and shown as a relative mRNA level. Data are shown as mean + SD. Three mice were used for each group. ns, not significant; *, P<0.05; ***, P<0.001. (PDF) [file ppat.1003478.s008.pdf]

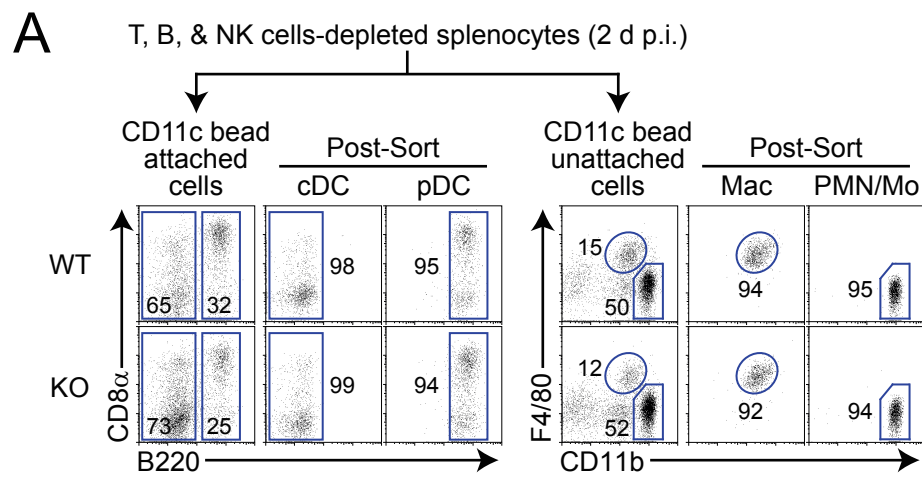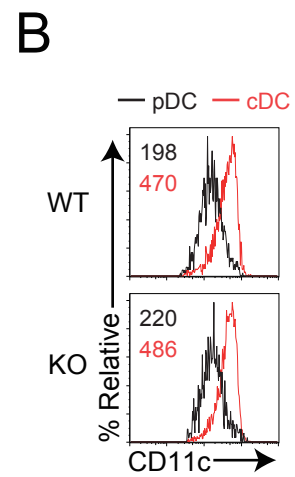

Supplement: Figure S9 — Splenic cell sorting strategy and post-sort verification of purity. (A) To obtain homogenous populations for splenic conventional DCs (cDC) and plasmacytoid DCs (pDC) (left panel), and for splenic macrophages (Mac) and polymorphonuclear cells/monocytes (PMN/Mo) (right panel), the splenocytes from LCMV CL-13-infected WT and Oasl1 KO mice (pooled from at least 10 mice per group) were isolated at 2 d p.i. and sorted by using flow cytometry. The purified cells were then re-analyzed for purity check. (B) CD11c expression level of the sorted pDC (black line) and cDC (red line) from WT and Oasl1 KO mice. Numbers in the plot indicate MFI value of CD11c expressed by pDC (black letter) and cDC (red letter). (PDF) [file ppat.1003478.s009.pdf]

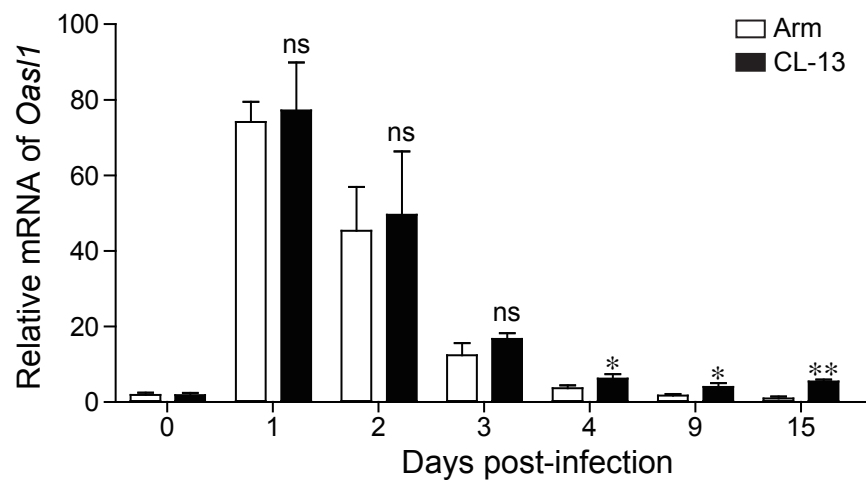

Supplement: Figure S10 — Comparison of Oasl1 mRNA expression levels in the spleen after infection with LCMV Arm or CL-13. WT mice were uninfected (0 d) or infected with the same dosage (2×10^6^ PFU/mice) of LCMV Arm or CL-13 and via the same route and their spleens were collected at the indicated time points p.i. Oasl1 mRNA expression level normalized to Gapdh mRNA amount was recalculated by dividing each expression value with the least mRNA expression value among the samples. Data are representative of two independent experiments (n = 3 per group in each experiment) and shown as mean + SD. ns, not significant; *, P<0.05; **, P<0.01. (PDF) [file ppat.1003478.s010.pdf]

A

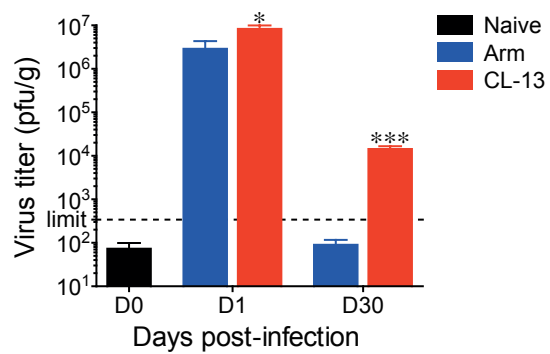

B

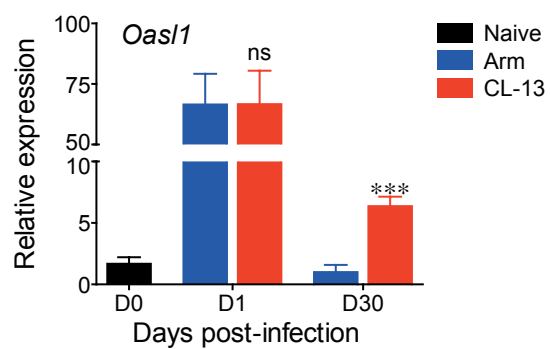

C

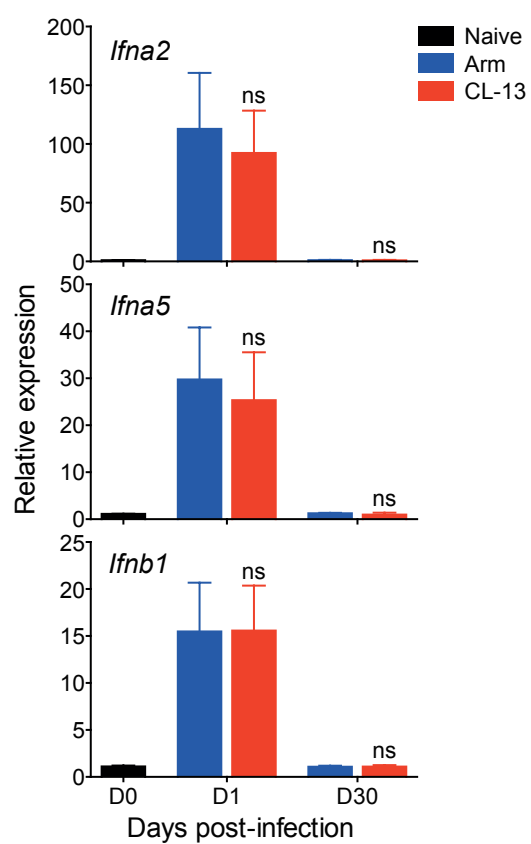

Supplement: Figure S11 — Gene expression levels of Oasl1 and Ifn-I on pDCs at 1 and 30 d p.i. WT B6 mice were infected i.v. with 2×10^6^ PFU of LCMV Arm or CL-13. Spleens were obtained from naive (0 d p.i.) and virus-infected mice 1 d and 30 d p.i. Single cell-suspended splenocytes were depleted of T cells, B cells, NK cells, and granulocytes by bead depletion. After enrichment of splenic CD11c+ cells using CD11c beads, the cells stained with anti-CD11c, CD8a, and B220 Abs were sorted to obtain plasmacytoid DCs using the strategy shown in Figure S9. (A) Virus titers in the spleens. At each time point, spleens obtained from naïve, Arm-, and CL-13-infected mice were homogenized to measure virus titers. Dashed black line represents the virus detection limit. (B, C) Relative mRNA expression levels of Oasl1 (n = 3 per group) (B) and Ifn-I (C) including Ifna2, Ifna5, and Ifnb1 on pDC at 0, 1, and 30 d p.i. All bar graphs show mean + SD. ns, not significant; *, P<0.05; ***, P<0.001. (PDF) [file ppat.1003478.s011.pdf]
